# Optimizing Biocompatibility and Gene Delivery with DMAEA and DMAEAm: A Niacin-Derived Copolymer Approach

**DOI:** 10.1021/acs.biomac.4c00007

**Published:** 2024-07-04

**Authors:** Prosper P. Mapfumo, Liên S. Reichel, Thomas André, Stephanie Hoeppener, Lenhard K. Rudolph, Anja Traeger

**Affiliations:** † Institute of Organic and Macromolecular Chemistry (IOMC), Friedrich Schiller University Jena, Humboldtstrasse 10, Jena 07743, Germany; ‡ Jena Center for Soft Matter (JCSM), Friedrich Schiller University Jena, Philosophenweg 7, Jena 07743, Germany; § Leibniz Institute on Aging-Fritz Lipmann Institute, Jena 07745, Germany

## Abstract

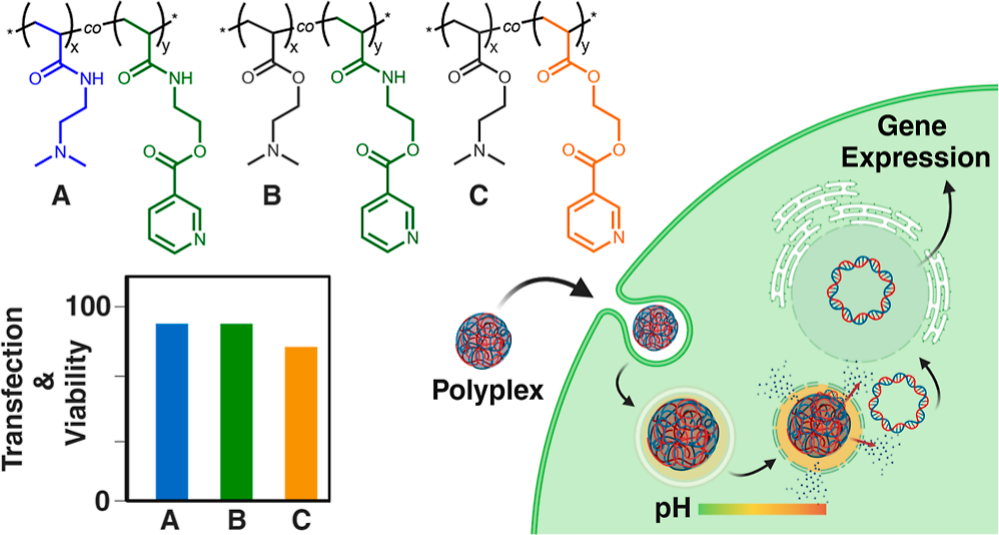

Gene therapy is pivotal in nanomedicine, offering a versatile approach to disease treatment. This study aims to achieve an optimal balance between biocompatibility and efficacy, which is a common challenge in the field. A copolymer library is synthesized, incorporating niacin-derived monomers 2-acrylamidoethyl nicotinate (AAEN) or 2-(acryloyloxy)ethyl nicotinate (AEN) with *N,N*-(dimethylamino)ethyl acrylamide (DMAEAm) or hydrolysis-labile *N,N*-(dimethylamino)ethyl acrylate (DMAEA). Evaluation of the polymers’ cytotoxicity profiles reveals that an increase in AAEN or DMAEA molar ratios correlates with improved biocompatibility. Remarkably, an increase in AAEN in both DMAEA and DMAEAm copolymers demonstrated enhanced transfection efficiencies of plasmid DNA in HEK293T cells. Additionally, the top-performing polymers demonstrate promising gene expression in challenging-to-transfect cells (THP-1 and Jurkat cells) and show no significant effect on modulating immune response induction in *ex vivo* treated murine monocytes. Overall, the best performing candidates exhibit an optimal balance between biocompatibility and efficacy, showcasing potential advancements in gene therapy.

## Introduction

In nanomedicine, gene therapy has emerged as a key treatment method due to its high potential and adaptable mechanism for treating incurable diseases such as autoimmune diseases, genetic disorders, and cancer.^1,2^ It involves the transient or stable expression of exogenous nucleic acids, such as small interfering RNA (siRNA), mRNA (mRNA), and plasmid DNA (pDNA), within specific cells or tissues.^3,4^ Introduction of nucleic acids into specific cells or targeted areas demands a carrier system because of the swift extracellular nuclease degradation and the potential to induce nonspecific immune reactions.^5,6^ In the case of siRNA or mRNA expression, the release from the complex should take place in the cell cytoplasm, while pDNA requires access to the nucleus. Consequently, among other factors, delivering nucleic acids, especially pDNA, presents a relatively greater challenge.^7−9^ Although viruses have demonstrated success in delivering pDNA and other nucleic acids, as evident from numerous approved clinical trials, concerns about their capacity to trigger undesirable immune responses persist.^10^ To surmount these challenges, approaches involving nonviral carriers hold great promise.^10,11^

To this end, lipids have emerged as the most promising among nonviral carriers, as underscored by their recent success in facilitating lipid nanoparticle–mRNA vaccines.^7,12^ Nonetheless, while they have proved to be effective at delivering siRNA or mRNA, their capacity to effectively deliver pDNA remains constrained.^10,12^ As a result, polymer-based delivery systems have emerged as an alternative technology that brings additional advantages in terms of robustness, reproducibility, scalability, and adaptability.^13^ However, the advancement of polymer-based strategies into translational research has been hindered by a pivotal challenge: striking the right balance between toxicity and efficacy, as researchers contend with the task of designing polymers that exhibit minimal toxicity while maximizing their efficacy.^10,13^ In brief, a successful gene carrier system should (i) be biocompatible, (ii) bind and protect nucleic acids from extracellular degradation and serum interaction, (iii) ensure safe transport and targeted uptake into specific cell types, (iv) exhibit high transfection profiles without deleterious unwanted reactions, and (v) be inexpensive and reproducible.^14−17^

Advances in synthetic methodologies, including controlled radical polymerization, such as reversible addition–fragmentation chain-transfer (RAFT), have facilitated the synthesis of polymers with tailored compositions and complex architectures.^18−21^ This becomes particularly advantageous when designing a carrier to meet specific requirements. In recent research, poly[*N,N*-(dimethylamino)ethyl acrylate] (PDMAEA) has emerged as a highly promising polymeric gene carrier due to its charge-shifting capabilities.^22−24^ PDMAEA self-hydrolyzes, transitioning from a predominantly cationic polymer to one with a more anionic backbone.^25,26^ The resulting nontoxic byproducts reduce the overall polymer toxicity.^26^ Additionally, this property of charge-shifting enables the controlled release of the genetic payload during gene delivery.^27^ For example, Truong *et al.* observed enhanced transfection efficiencies with higher molecular weight PDMAEA.^27^ However, the charge-shifting property also contributes to the low success of PDMAEA, as hydrolysis can occur faster than the delivery of genetic material, resulting in the loss of the payload.^27^ Interestingly, a study of the hydrolytically stable acrylamide variant, poly[*N,N*-(dimethylamino)ethyl acrylamide] (PDMAEAm), showed that the polymer exhibits low transfection efficiencies across various molecular weights, albeit with enhanced cell internalization.^28^

To this end, to enhance the delivery performance of cationic polymers, one approach involves the incorporation of hydrophobic components.^29−32^ Hydrophobic components enhance complex stability, protect against serum interactions, and promote membrane interactions for improved cellular uptake and potential endosomal release mechanisms.^33−36^ However, the selection of the hydrophobic moieties is important because they can induce cytotoxicity, even though transfection performance is improved.^36−38^ For example, in our previous work, a positive correlation between cytotoxicity, transfection performance, and pendant acyclic alkyl chain length was observed. In contrast, an increase in lipoic acid-derived monomer reduced cytotoxicity and boosted transfection performance.^38^ This result highlighted the potential of incorporating nutrient-derived components to improve performance. To elaborate further, hydrophobic homopolymers derived from niacin were investigated.^39^ The rationale was to utilize niacin, a natural compound, to improve nanoparticle formation, biocompatibility, and uptake profiles of polymers by harnessing the intrinsic advantages of the natural compound. Poly[2-acrylamidoethyl nicotinate] (PAAEN) and poly[2-(acryloyloxy)ethyl nicotinate] (PAEN) emerged as the top performers, with their nanoparticles showcasing high biocompatibility and enhanced uptake profiles, making them promising candidates for gene delivery if their attributes can be retained.^39^

In this study, we investigate a copolymer library comprising niacin-derived monomers (AAEN or AEN) in combination with either DMAEAm or DMAEA. The latter is incorporated to harness its charge-shifting property and compare its performance to that of the hydrolytically stable DMAEAm. Furthermore, we aim to retain and utilize the high uptake profiles of niacin-derived moieties to enhance the overall delivery performance of the polymers.

## Experimental Section

The Supporting Information contains additional details about the experimental specifics of materials and instrumentation, as well as the procedures used for monomer synthesis and biological investigations. Monomer and polymer characterizations were carried out by nuclear magnetic resonance (NMR) spectroscopy and size-exclusion chromatography (SEC) (Supporting Information; Figure S1 shows ^1^H and ^13^C NMR spectra of monomers. Figures S2 and S3: ^1^H NMR and SEC plots of all polymers).

### Polymer Synthesis

The quantities of monomers used for synthesis and their conversions are further detailed in the Supporting Information, Table S1.

#### Synthesis of P(AAEN_148/123/48_-*co*-DMAEAm_50/75/142_) (**A1–A3**)

(Propionic acid)yl butyl trithiocarbonate (PABTC) (10.0 mg, 4.19 × 10^–5^ moles), DMAEAm, AAEN, dioxane (4.3 g), a 1 wt % solution of 2,2′-azobis(2,4-dimethylvaleronitrile) (V-65) in dioxane (0.95 g, 9.25 mg V-65b, 3.58 × 10^–5^ moles), and 1,3,5-trioxane (external NMR standard) (28–30 mg) were, respectively, introduced to a 8 mL microwave vial equipped with a magnetic stirring bar. The vial was sealed, and the solution was deoxygenated by bubbling argon through it for 10 min. Afterward, the vial was placed in an oil bath at 50 °C and allowed to stir for 4.5 h. Samples were taken prior to the start of the reaction and after the reaction was stopped. The polymers were precipitated three times into cold ether, and each time they were redissolved in methanol (MeOH). Finally, the polymers were dried under reduced pressure to give a yellowish solid.

#### Synthesis of P(AAEN_152/120/49_-*co*-DMAEA_50/72/149_) (**B1–B3**)

PABTC (10.0 mg, 4.19 × 10^–5^ moles), DMAEA, AAEN, dioxane (3.9 g), a 0.5 wt % solution of 4,4′-azobis(4-cyanovaleric acid) (ACVA) in dioxane (344.0 mg, 1.72 mg ACVA, 6.13 × 10^–6^ moles), and 1,3,5-trioxane (external NMR standard) (26–29 mg) were, respectively, introduced to a 8 mL microwave vial equipped with a magnetic stirring bar. The vial was sealed, and the solution deoxygenated by bubbling argon through it for 10 min. Afterward, the vial was placed in an oil bath at 70 °C and allowed to stir for 23 h. Samples were taken prior to start of reaction and after reaction was stopped. The polymers were precipitated three times into cold ether, redissolving it each time in chloroform (CHCl_3_) for **B1** and **B2** and tetrahydrofuran (THF) for **B3**. Finally, the polymers were dried under reduced pressure to give a yellowish solid.

#### Synthesis of P(AEN_115_-*co*-DMAEA_65_) (**C1**)

PABTC (10.0 mg, 4.19 × 10^–5^ moles), DMAEA (0.54 g, 3.78 × 10^–3^ moles), AEN (1.48 g, 6.71 × 10^–3^ moles), dioxane (4.6 g), a 0.5 wt % solution of ACVA in dioxane (764.0 mg, 3.82 mg ACVA, 1.36 × 10^–5^ moles), and 1,3,5-trioxane (external NMR standard) (29 mg) were, respectively, introduced to a 8 mL microwave vial equipped with a magnetic stirring bar. The vial was sealed, and the solution deoxygenated by bubbling argon through it for 10 min. Afterward, the vial was placed in an oil bath at 70 °C and allowed to stir for 15 h. Samples were taken prior to start of reaction and after reaction was stopped. The polymer was precipitated three times into cold ether, with the solution redissolved each time in CHCl_3_. Lastly, the polymer was dissolved in MeOH and to this was added 582 μL of 4 M HCl in dioxane, and the solution was precipitated into cold ether and dried under reduced pressure to give a yellowish solid.

#### Titrations

Titrations of the polymers were conducted by using a Metrohm OMNIS integrated titration system. For a typical measurement, 200 mg of polymer was dissolved in 0.2 M HCl (10 mL), and an additional 1 equiv of HCl to the polymer amine groups was added. The polymers were titrated (with dynamic flow rate adjustment) against a 0.15 M NaOH solution.

#### DMAEA Copolymer Degradation

The polymers used for the investigation were HCl salts; 1 equiv of HCl to the DMAEA components was added and dried. In a standard measurement, 30 mg of the copolymers and 26 mg for PDMAEA_125_ were dissolved in D_2_O, followed by the addition of 200 mM NaOH in D_2_O (Supporting Information. Added amounts used are provided in Table S2. The resulting pH for all samples was approximately 7.5. The ^1^H NMR sample was measured and subsequently incubated at 37 °C for 24 h (Supporting Information; Figure S6: ^1^H NMR spectra analyzing the hydrolysis of DMAEA containing polymers). After incubation, a second measurement was taken, and the degree of degradation was calculated based on the ^1^H NMR results using previously reported formulas shown in eqs S3 and S4.^26^

### Biological Assays

#### Cell Culture

The mouse fibroblast cell line L929 and human embryonic kidney cell line HEK293T were cultivated in Dulbecco’s modified Eagle’s medium (DMEM, 1 g L^–1^ glucose), supplemented with 10% (v/v) fetal bovine serum (FBS), 100 U mL^–1^ penicillin, and 100 μg mL^–1^ streptomycin (D10). The human monocytic cell line (THP-1) and the human T-lymphocyte cell line (Jurkat) were cultivated in RPMI 1640 with Stable Glutamine medium (RPMI), supplemented with 10% (v/v) FBS, 100 U mL^–1^ penicillin, and 100 μg mL^–1^ streptomycin (R10). All cell lines were cultivated at 37 °C in a humidified 5% (v/v) CO_2_ atmosphere.

For PrestoBlue assay, L929 cell line was seeded at a cell concentration of 0.1 × 10^6^ cells mL^–1^ in a 96-well plate in a total volume of 100 μL D10, supplemented with 10 mM HEPES buffer (D10H) per well.

For transfection efficiency studies in HEK293T cells, 24 h before, the cells were seeded in a 24-well plate at a cell concentration of 0.2 × 10^6^ cells mL^–1^ in 500 μL of D10H to reach a cell confluency > 70%. One hour before the experiment started, the medium was changed to fresh D10H.

For transfection efficiency studies in THP-1 and Jurkat cell lines, the cells were seeded in a 24-well plate at a cell concentration of 0.3 × 10^6^ cells mL^–1^ in 500 μL of R10, supplemented with 10 mM HEPES buffer (R10H) 3 h before the transfection efficiency studies.

#### Cytocompatibility (PrestoBlue Assay)

The cytotoxicity of the polymers was conducted by determining the metabolic activity of viable cells. The PrestoBlue assay was performed based on ISO10993-5 with L929 cells.^40^ The medium was changed to 90 μL of fresh D10H 1 h before treatment. To determine the cytocompatibility in THP-1 and Jurkat cells, the cells were seeded 3 h before treatment. In triplicate, cells were treated with 10 μL of polymers, which were diluted in 20 mM sodium acetate buffer (NaOAc buffer, pH 5.4). The tested polymer concentrations were ranging from 15 to 500 μg mL^–1^ and 3 to 100 μg mL^–1^ for linear polyethylenimine (LPEI) as positive control for the assay. After 24 h of incubation, the medium was replaced by a 10% (v/v) PrestoBlue solution in fresh D10 and prepared according to the manufacturer’s instructions for the L929 cell line. For THP-1 and Jurkat cell lines, 10 μL of PrestoBlue solution was added directly to the medium. Cells were further incubated for 45 min at 37 °C before the fluorescence was measured with the multiplate reader at λ_Ex_ = 570/λ_Em_ = 610 nm. The NaOAc buffer-treated cells on the same plate were defined as having 100% viability. The relative number of viable cells was calculated as in eq 1
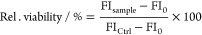
1where FI_sample_, FI_0_, and FI_Ctrl_ represent the fluorescence intensity of a given sample, medium without cells (the blank), and NaOAc buffer-treated control (100% viability), respectively.

#### Polymer–Membrane Interaction

To investigate the polymer–cellular membrane interaction, human erythrocytes were used. Blood from three different human donors preserved with EDTA additive was obtained from the Department of Transfusion Medicine of the University Hospital, Jena. To purify the erythrocytes, the blood was centrifuged without pooling at 4500 *g* for 5 min, and the supernatant (the serum) was removed. The pellet of erythrocytes was washed three times with cold phosphate-buffered saline (PBS, pH 7.4) and resuspended 10-fold with PBS (pH 7.4). The tested polymers were diluted with PBS (pH 7.4) to the aimed concentrations, ranging from 10 to 200 μg mL^–1^. Subsequently, 350 μL aliquots of erythrocyte suspension were mixed 1:1 (v/v) with the polymer solutions. The erythrocyte-polymer suspensions were incubated at 37 °C for 60 min and centrifuged at 2400*g* for 5 min before the supernatant was transferred in triplicate to a clear flat-bottomed 96-well plate. The hemoglobin release was determined as the hemoglobin absorption at λ = 544 nm. Absorption at λ = 630 nm was used as a reference. Complete hemolysis (100%) was achieved using 1% Triton X-100 as the positive control, since Triton X-100 strongly disrupts the cell membrane. Pure PBS was used as the negative control (0% hemolysis). The hemolytic activity of the polymer was calculated as follows
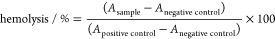
2where *A*_sample_, *A*_negative control_, and *A*_positive control_ are the absorption values of a given sample, the PBS treatment, and the Triton X-100 treatment, respectively. *A* value less than 2% hemolysis rate was classified as nonhemolytic, 2 to 5% as slightly hemolytic, and values > 5% as hemolytic.

#### Polyplexation

Plasmid DNA (pDNA) was diluted in 5% glucose supplemented with 20 mM HEPES buffer (HBG) to have a master mix with a pDNA concentration, which was twice as high as the final polyplex solution. The polymers were diluted in 20 mM NaOAc buffer at double the concentration as aimed in the final N*/P ratio (molar ratio of DMAEA/DMAEAm amines in the polymer to phosphates in pDNA). The master mix was added in a 1:1 (v/v) ratio to the diluted polymer solution. Immediately, the mixture was vortexed for 10 s at a maximum speed and further incubated for 15 min at room temperature.

#### Size Determination *via* Dynamic Light Scattering

Polyplex, prepared as described, was further investigated for its hydrodynamic diameter using dynamic light scattering (DLS Zetasizer Nano ZS). 70 μL of the polyplexes with the EGFP-noncoded plasmid pKMyc was used. Each sample was measured at 25 °C after an equilibration time of 30 s, and 15 size runs were performed with 0.839 s per run. The counts were detected at an angle of 173°. Water was used as a dispersant with a viscosity of 0.8872 mPa·s (at 25 °C) and a refractive index of 1.33. The mean particle size was approximated as the effective (z-average) diameter, and the distribution width was approximated as the polydispersity index (PDI) of the particles. General purpose was used as the analysis model, assuming a spherical shape of the polyplexes. Data was analyzed using ZS Xplorer software.

#### Interaction of Polymers and Genetic Material

Ethidium bromide (EtBr) binding assay (EBA) and heparin release assay (HRA) were conducted to investigate the binding affinity and release ability between the polymer and genetic material. The assays are based on measuring the increased or decreased fluorescence intensity of EtBr when intercalating with the genetic material or release of genetic material, respectively.^41^ To study the ability of the polymer to complex genetic material, an EBA was performed. Therefore, the pKMyc was diluted as described in the polyplexation section with addition of EtBr (1 μg mL^–1^). The solution was incubated and protected from light at RT for 10 min. The polymers were diluted with HBG buffer (pH 7.4) to give an N*/P ratio from 1 to 20. Subsequently, the master mix-EtBr solution was added 1 to a 1:1 volume ratio with the different polymer solutions using black 96-well plates. The solution was mixed by resuspension and incubated at 37 °C for 15 min. The fluorescence intensity was measured at λ_Ex_ = 525 nm/λ_Em_ = 605 nm. As maximum fluorescence (100%), a sample containing only pKMyc and EtBr was used as the control. To investigate the ability to release genetic material from the polyplex, heparin, a polyanion, was added to the polyplexes at different concentrations using the dispenser of the microplate (Supporting Information; added amounts are shown in Table S3). After each addition, the plate was shaken and incubated for 10 min before determining the fluorescence intensity.

The percentage of EtBr displaced due to polyplex formation or reintercalating following pDNA release by heparin was calculated in the following eq 3
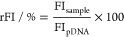
3rFI is the relative fluorescence intensity, FI_sample_ and FI_pDNA_ are the fluorescence intensities of the sample, and the EtBr is intercalated into pDNA only (100%), respectively.

#### Cryogenic Transmission Electron Microscopy (Cryo-TEM)

The samples for cryo-TEM were prepared as described in the polyplexation section. Cryo-TEM images were acquired with an FEI Tecnai G^2^ 20 at an acceleration voltage of 120 kV with an Olympus MegaView camera (1379 × 1024 pixels). Sample preparation was performed by plunge-freezing the samples with a Vitrobot Mark IV system. 9.0 μL of the aqueous solutions were blotted on Quantifoil grids (R2/2, Quantifoil, Jena, Germany) and were vitrified in liquid ethane. The grids were rendered hydrophilic by Ar-plasma cleaning for 30 s (Diener Electronics, Germany) prior to the sample preparation process. After vitrification, samples were stored in liquid nitrogen until transferred to the cryo-holder (Gatan 626). Transfer to the microscope was performed with a Gatan cryo-stage, and the temperature was always maintained below −172 °C after vitrification.

#### Particle Uptake

HEK293T cells were seeded as described in the cell culture section. One hour before treatment, the medium was replaced with fresh 45 μL of D10H medium. The polyplexes were prepared as previously described in the polyplexation section but with the addition of YOYO-1 to the master mix. Cells were treated with 50 μL polyplex at N*/P 20 with a final concentration of 3 μg mL^–1^ EGFP-noncoded plasmid pKMyc on cells over 1 and 4 h. After the incubation, cells were harvested by adding 150 μL of Trypsin EDTA and incubating them for 10 min at 37 °C (5% CO_2_). Following, 350 μL of fresh D10 was added to stop trypsinization, and 250 μL of cell suspension was transferred to a 96-well plate for flow cytometry analysis. For detection, a bandpass detection filter 525 ± 40 nm was used.

#### Transfection Efficiency

HEK293T cells were seeded as described in the cell culture section. One hour before treatment, the medium was replaced with fresh 45 μL D10H medium. The polyplexes were prepared as previously described in the polyplexation section. Cells were treated with 50 μL polyplex at N*/P 20 with a final pDNA concentration of 3/2/1 μg mL^–1^ on cells or with 50 μL polyplex at N*/P 20/10/5/3 and a final pDNA concentration of 3 μg mL^–1^ on cells over 24 h. After 24 h incubation, 50 μL of the supernatant was transferred to a 96-well plate in triplicate for membrane integrity investigation. After the incubation, the supernatant was used to conduct the cytocompatibility of polyplexes (CytoTox-One assay), and cells were harvested as described in the particle uptake section.

To investigate the transfection in hard-to-transfect cell lines THP-1 and Jurkat, cells were seeded 3 h before treatment, as described in the cell culture section. Cells were treated with 50 μL polyplex at N*/P 20 and a final pDNA concentration of 3 μg mL^–1^ on cells over 24 h. Then 250 μL of the cell suspension was transferred to a U-bottom 96-well plate. Cells were washed with centrifugation (1000 *g*, 5 min) twice with PBS (pH 7). For flow cytometry, cells were resuspended in 250 μL of PBS (pH 7), and a bandpass detection filter 510 ± 10 nm with signal attenuation (OD1) was used.

#### Cytocompatibility of Polyplexes (CytoTox-ONE Assay)

To determine the membrane integrity of polyplex-treated HEK293T cells for transfection efficiency studies, the CytoTox-ONE assay was performed. After incubation with the polyplexes, 50 μL of the supernatant was transferred to a 96-well plate in triplicate and was equilibrated for 20 min to reach room temperature. Following, 50 μL of CytoTox-ONE reagent was added to each well. After 10 min of incubation at room temperature, 25 μL of stop solution was added. Cells treated with lysis solution were used as a 100% control. Values lower than 90% viability were regarded as being cytotoxic. The fluorescence intensity was measured at λ_Ex_ = 570/λ_Em_ = 610 nm, and cytotoxicity was calculated as follows
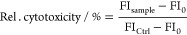
4where FI_sample_, FI_0_, and FI_Ctrl_ represent the fluorescence intensity of a given sample, medium without cells (the blank), and lysis-treated cells (100% cytotoxicity), respectively.

#### Endosomal Release

To study the endosomal release, HEK293T cells were seeded at 0.2 × 10^6^ cells mL^–1^ in an 8-well chamber slide. The cells were preincubated for 24 h in D10H. One hour before treatment, old medium was replaced by 225 μL of new D10H. Cells were treated with calcein (25 μg mL^–1^) followed by treatment with the polyplexes (N*/P 20, 3 μg mL^–1^ pDNA). After 4 h incubation, cell nuclei were stained with Hoechst 33342 for 5 min and washed twice with warm Hanks’5 balanced salt solution with addition of 2% serum. For imaging, cells were further incubated in full growth medium with 10% FCS (D10).

Cell images were captured using a confocal laser scanning microscope LSM880, Elyra PS.1 system (Zeiss, Germany).

#### *Ex Vivo* Monocyte Culture

Nonsuffering mice with a noninduced transgenic construct and a C57BL/6 background were used. All animals were kept in a specific pathogen-free animal facility with a 12 h light/dark cycle. Mice were killed according to international and national regulations with an increasing CO_2_ exposure. All mice were 4–5 months old.

The mice’s hind limbs (including hip bones joints), forelimbs, and spines were dissected, cleaned, and crushed in 2% FBS using mortar and pestle. After incubation for 5 min on ice with FcR-Blocking Reagent, bone marrow cells were incubated with 7 μL APC-conjugated anti-Ly-6C antibody for 30 min, and Ly-6C^+^ cells were enriched using anti-APC magnetic beads (MACS Miltenyi Biotec 130-09-855) and LS columns. Ly-6C positive cells were then stained with an antibody mix against CD11B, CD115, and Ly6C (Supporting Information; added amounts are provided in Table S5) for 60 min on ice. In addition, samples were stained 5 min before sorting with DAPI. 200.000 cells were sorted on an ARIA III cell sorter (BD bioscience) into one tube (for one well). Single cells being DAPI negative, CD11b^+^, CD115^+^, and Ly-6C^+^ (classical monocytes) were sorted. Cells were spun down, and supernatant was removed. Cells were resuspended in 900 μL monocyte culture medium and plated in 24-well plates. Cells were incubated at 37 °C, 5% CO_2_, and 95% humidity for 1 day. The next day, 100 μL of the following five chemicals were added (one per well), and incubation was continued for 24 h until RNA isolation: (1) NaOAc 20 mM buffer (pH 5.4) was used as a solvent for all following chemicals. (2) Niacin was solved and diluted to the same molarity corresponding to the niacin molarity in the polymer sample (1.09 mmol L^–1^). (3) Polymer **C1** was diluted to the same polymer concentration as that used in the polyplex (35 μg mL^–1^). (4) pDNA was diluted to 10 μg mL^–1^. (5) Polyplex (pDNA + **C1**) was assembled as described in the methodology for polyplexation with N*/P 20 and 10 μg mL^–1^ EGFP-pDNA to achieve a final concentration of 1 μg mL^–1^ EGFP-pDNA on cells. Lastly, the untreated group received nothing. One replicate (=biological replicate) per each condition represents one mouse donor, so that no condition has multiple replicates from the same mouse.

#### RNA Isolation and RT-qPCR

Cell culture supernatant was removed, and cells were washed once with DPBS. 1 mL portion of TRIzol (Invitrogen) was directly added onto the plate and incubated for 5 min at room temperature. RNA was isolated with 200 μL of chloroform by shaking for 15 s, centrifuging for 15 min at 16100 G, and then transferring the upper, aqueous layer to a new tube. RNA was then precipitated with 500 μL of isopropanol and 3 μL of 20 mg mL^-1^ glycogen overnight at −20 °C. RNA was washed twice with 75% ethanol, and DNA was digested with DNase for 20 min at 37 °C. DNase reaction was stopped with EDTA incubation for 10 min at 65 °C. RNA content was measured on a Nanodrop 2000c, Thermo Fisher. 200 ng of RNA was used for cDNA conversion using the GoScript Reverse Transcriptase Kit (Promega, Cat. No: A5001) following manufacturer’s recommendations. The RT-qPCR reaction was performed in a volume of 15 μL with 7.5 μL of iTaq Universal SBYR Green Supermix (BIO-RAD, cat. no: 1725124), 3 μL of 1:1 with water diluted cDNA, and 500 nm of each Primer and run on a BIO-RAD CFX384 Real-Time System machine in 384-well plates. The following RT-qPCR primers (all for mice) were used in this study: TNF-α sense 5′- GCC TCT TCT CAT TCC TGC TTG -3′, TNF-α antisense 5′- CTG ATG AGA GGG AGG CCA TT -3′, GAPDH-sense 5′- TCA TGG ATG ACC TTG GCC AG-3′, and GAPDH-antisense 5′- GTC TTC ACT ACC ATG GAG AAG G-3′. Each sample was run in duplicates. Mean Ct values were normalized against the GAPDH expression. Nuclease-free, DEPC-treated water was used in every step.

#### Statistical Analysis

Statistical analyses were calculated using OriginPro2022b for data of transfection efficiency assays. All data were first tested for normality with the Kolmogorov–Smirnov test. Before testing for normality, log transformation was calculated for the ratio of EGFP positive cells to total cell count. Following, one-way analysis of variance (ANOVA) was performed with Turkey posthoc test for MFI and the ratio of EGFP positive cells to total cell count.

Statistical testing for the RT-qPCR data was performed in GraphPad Prism version 9.0.2 (161). DeltaCt values were tested condition-wise onto normality with an Anderson–Darling, D’Agostino–Pearson, Shapiro–Wilk, and Kolmogorov–Smirnov test. A student-*t* test with Welch́s correction was performed on every comparison. *P*-values were corrected with a Holm-Šídák test. Results were considered significant on each test if *p* < 0.05.

## Results and Discussion

### Synthesis and Characterization

**Figure 1:**
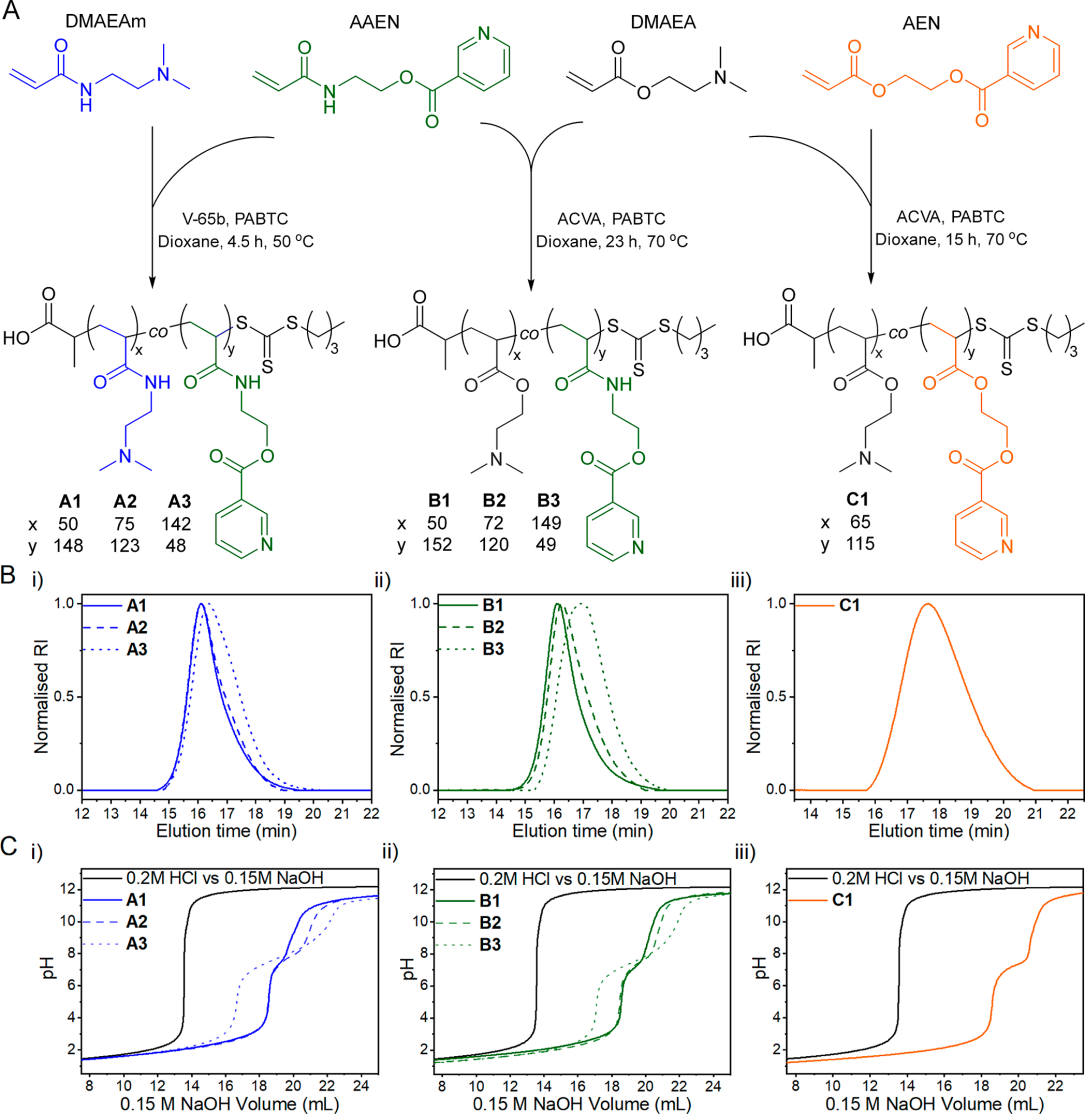
(A) Reaction scheme illustrates the structures of the polymer library and their polymerization conditions. (B) SEC traces of the polymer library using DMAc (0.21 wt % LiCl) as eluent. (C) Titration curves of the polymer library were determined using 0.15 M NaOH as the base with a Metrohm OMNIS integrated titration system.

In our previous study, PAAEN demonstrated superior attributes in both encapsulation and cellular uptake when compared to PAEN.^39^ Therefore, to further explore the potential applications of niacin-derived polymers as gene carriers, a copolymer library primarily centered on AAEN was synthesized with one additional polymer consisting of AEN (Figure 1A). The **A** and **B** polymer series, which were based on the more promising AAEN, *i.e.*, P(AAEN_148/123/48_-*co*-DMAEAm_50/75/142_) (**A1–A3**) and P(AAEN_152/120/49_-*co*-DMAEA_50/72/149_) (**B1–B3**), encompassed three distinct molar ratios each. The molar ratios between the polymer sets were comparable to facilitate comparisons of biological and polymer properties. On the other hand, the synthesis of P(AEN_115_-*co*-DMAEA_65_) (**C1**) was conducted following initial investigations into the transfection efficiency of the **A** and **B** series, which resulted in the identification and selection of the optimal ratio. Analysis of molar mass distributions of the polymers using SEC revealed monomodal distributions, as depicted in Figure 1B. In addition, the dispersities (D̵) of the molar mass distributions remained consistently at 1.3, except for **C1**, which showed a slight deviation with a value of 1.4, as outlined in Table 1. These findings collectively suggest a relatively effective control of the RAFT polymerization process. This was further substantiated by examining the polymerization kinetics of each copolymer composition (Supporting Information; plots monitoring polymerization kinetics are provided in Figure S4). In each composition, the kinetics exhibited a similar rate of polymerization for each monomer with conversions typically ranging from 70 to 80%. Moreover, the polymerization kinetic for each polymer set revealed similar monomer reactivities, which were reproducible during polymerization of the library (Supporting Information; monomer conversions are provided in Table S1). This suggests a statistical distribution of copolymers, even in the **B** set, which comprised two distinct functional groups (acrylamide and acrylate).

**Table 1:** Summary of the Molar Masses of the Polymer Library and the Apparent p*K*_a_ Values of DMAEAm or DMAEA

	**A1**	**A2**	**A3**	**B1**	**B2**	**B3**	**C1**
*M*_n,th_^a^ (kg mol^–^^1^)	40.1	38.0	31.1	40.8	37.0	32.3	34.9
*M*_n,SEC_^b^, (kg mol^–^^1^)	41.0	41.5	33.2	40.5	36.8	26.5	14.7
D̵^b^	1.3	1.3	1.3	1.3	1.3	1.3	1.4
p*K*_a_^c^DMAEAm/DMAEA	7.6	7.8	8.0	7.2	7.5	7.7	7.3

a Calculated *via* conversion using ^1^H NMR and eq S1.

b Determined by SEC using DMAc (0.21 wt % LiCl) as eluent and PMMA standards for calibration.

c Determined using degree of charge (DoC) plots, which were calculated using eq S2 (Supporting Information; DoC and titration plots of all polymers are provided in Figure S5).

Following polymerization, titration experiments were conducted to determine the apparent p*K*_a_ values of the polymers. The relevance of this parameter stems from the correlation between p*K*_a_ and efficacy of carriers.^42,43^ During these titrations, all polymers exhibited a tendency to precipitate from solution due to their hydrophobic nature. As shown in Figure 1C, the buffering regions for DMAEAm and DMAEA displayed a plateau, expanding as their molar ratios increased. The calculated p*K*_a_ values for these tertiary amines ranged between pH 7.2 and 8.0, as summarized in Table 1. When comparing the similar molar ratios between the polymer series, it was evident that the p*K*_a_ values for the **A** series consistently exceeded those of the corresponding **B** series, underscoring the influence of hydrophobicity in reducing p*K*_a_ values. This effect was further displayed when comparing polymers **A2**, **B2**, and **C1**, which shared similar ratios and exhibited a decreasing p*K*_a_ in that respective order with an increase in hydrophobicity.

Notably, an intriguing buffering region spanning approximately pH 1.8 to 3.8 was observed for all polymers, as illustrated in Figure 1C. The buffering range decreased noticeably as the niacin content decreased, a trend particularly evident when comparing polymers **A3** and **B3** with their respective counterparts. Therefore, this buffering range could be attributed to the nitrogen of niacin. Since the nitrogen in niacin has a known p*K*_a_ value of 4.8,^44^ it was postulated that the hydrophobic nature of the polymers led to the lowering of the p*K*_a_ value. Consequently, the p*K*_a_ of niacin within the copolymers was estimated to be in a range of 2.5–3.0.

Since PDMAEA is known for its self-hydrolysis and previous studies have explored its behavior under various conditions,^27,45^ an in-depth study on its hydrolysis was not conducted. Nevertheless, the stability of **A2**, **A3**, and PDMAEA_125_ was investigated by ^1^H NMR at physiological pH, over 24 h at 37 °C, a condition relevant to transfection studies. **A1** and **C1** were excluded due to poor solubility at similar pH. Subsequent analysis indicated a 52% hydrolysis rate for **A2** and a 43% hydrolysis rate for both **A3** and PDMAEA_125_ (Supporting Information; ^1^H NMR spectra analyzing the hydrolysis of DMAEA containing polymers are shown in Figure S6). The slightly elevated level of hydrolysis of **A2** was attributed to its lower p*K*_a_ value, resulting in fewer protonated DMAEA moieties and, consequently, more hydrolysis.

### Polyplex Formation and Characterization

**Figure 2:**
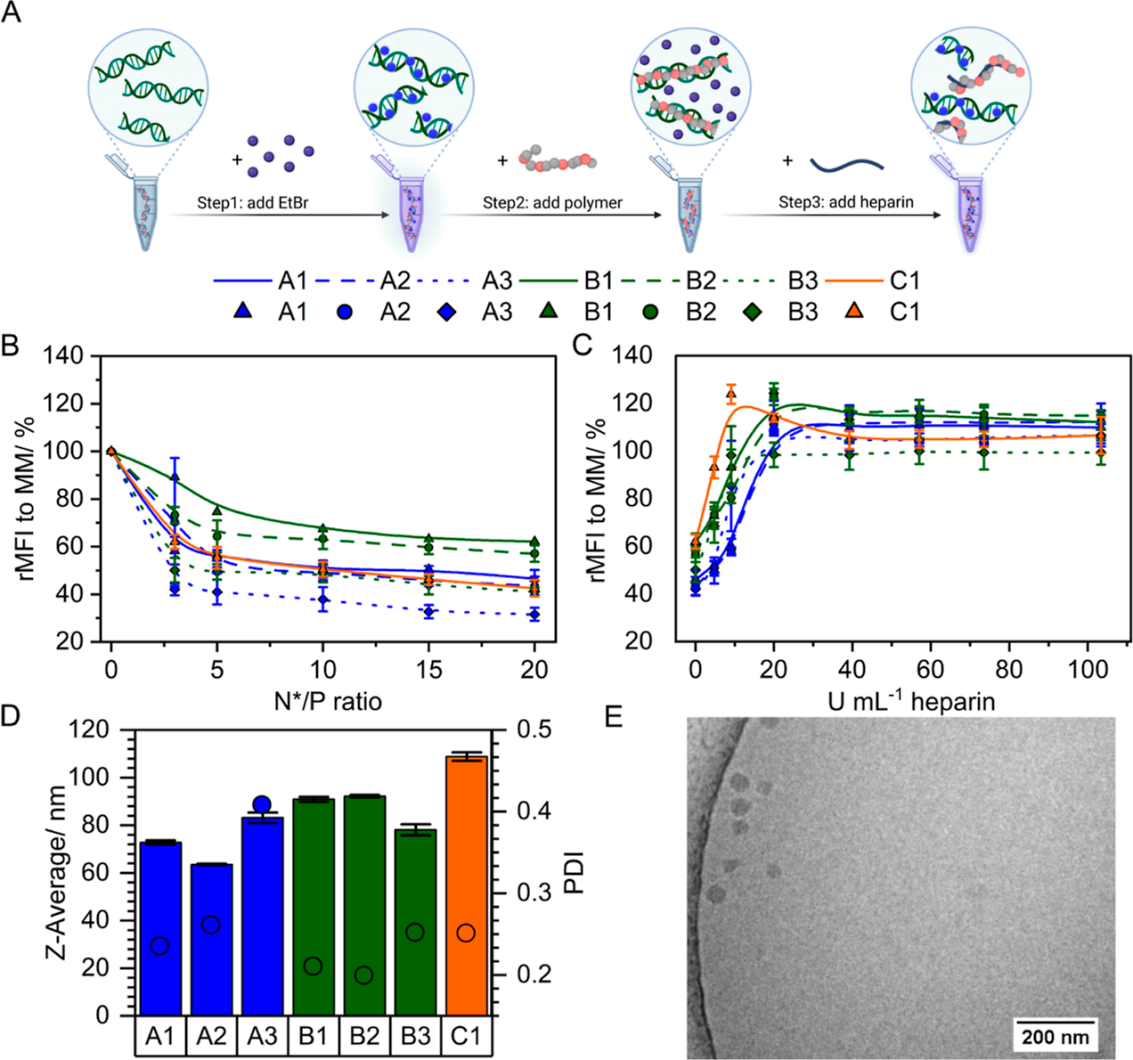
(A) Experiment design of EBA (first two steps) and HRA (all three steps). (B) EBA was performed at different N*/P ratios ranging from 3 to 20. (C) HRA assay at N*/*P* = 20. All data points were performed in triplicate, and values were fitted using a B-Spline function. (D) Hydrodynamic size measurement *via* DLS. (E) Cryo-TEM image of polyplexes of **C1**.

To assess the polymers’ ability to bind and release pDNA, fluorescence-based assays (EBA and HRA) were utilized (Figure 2A). These assays characterize the polymer’s complexation potential with pDNA by measuring the fluorescence intensity changes of EtBr, an intercalating dye. In EBA, polyplex formation displaces EtBr from pDNA into the aqueous environment, resulting in a decreased signal intensity. Conversely, in HRA, heparin competes for the cationic polymer within the polyplex, leading to the release of pDNA, followed by its reintercalation with EtBr in the solution, causing an increase in signal intensity.^46,47^ Initially, EBA was conducted for all polymers at varying N*/P ratios (3 to 20). Figure 2B shows a decrease in fluorescence intensity due to EtBr displacement from pDNA for all polymer polyplexes. The decrease correlates with an increase in the N*/P ratio, indicating an enhanced polyplex formation. Consequently, N*/P 20 was selected as the optimal ratio. Interestingly, **A** polymers exhibited improved binding capacity compared to **B** polymers and **C1**. Conversely, in HRA at N*/P 20 (Figure 2C), **C1** released pDNA at lower heparin concentrations compared to those of the **B** and **A** polymers, respectively. Approximately, 20 U mL^–1^ of heparin was required to completely release pDNA for all polymers, which is consistent with previous findings from our research group.^33,48^ Further results of different N*/P ratios can be found in the Supporting Information, Figure S7.

These observations are mainly due to two factors: (i) electrostatic interactions between the cationic polymer and anionic pDNA, and (ii) hydrogen bonding between polymer moieties, such as the amide backbone and pDNA. Although other factors also contribute, these have been previously demonstrated as crucial in enhancing binding affinity.^49^ This is exemplified by the improved binding capacity observed in the order of **A1** to **A3** and **B1** to **B3**, respectively, as depicted in Figure 2B. It is worth noting that electrostatic interactions appear to be more dominant as the binding improves with an increase in cationic moieties (DMAEAm/DMAEA) and a decrease in backbone amide bonds. However, when comparing the performance of **B** polymers to **A** polymer series, the impact of hydrogen bonding is emphasized, as substituting amide bonds with ester bonds leads to a decrease in efficient binding. Nevertheless, a contrasting trend was observed for pDNA release, with **C1** demonstrating the easiest release, followed by **B** polymers and then **A** polymers, respectively. This pattern emerged because enhanced binding corresponded to a relatively lower tendency to release pDNA, as depicted in Figure 2C. Surprisingly, **C1** exhibited improved binding affinity compared to **B1** and **B2**. This was hypothesized to be due to increased hydrophobicity and a low glass transition temperature (*T*_g_) of **C1** (6 °C) compared to **B1** (69 °C) and **B2** (52 °C) (Supporting Information; differential scanning calorimetry plots are provided in Figure S8). A lower *T*_g_ indicates more flexible chains, potentially promoting electrostatic entanglement with the pDNA.^50,51^ However, the low *T*_g_ also potentially results in reduced particle stability, leading to a faster release of pDNA, as observed in Figure 2C. A similar tendency to lose cargo due to *T*_g_ was observed in our previous study on PAEN with small molecular weight model drugs.^39^

Since cellular internalization of polyplexes is strongly influenced by their size,^52^ the sizes of polyplexes for all polymers formulated at N*/P 20 were determined using DLS. As shown in Figure 2D, the hydrodynamic diameters of the polyplexes ranged between 64 and 109 nm, which is suitable for controlled cellular uptake through endocytosis.^52,53^ Interestingly, when comparing the hydrodynamic diameters of **A2** (64 nm), **B2** (92 nm), and **C1** (109 nm), which consist of similar molar ratios, a slight and gradual increase in the hydrodynamic diameter was evident. This increase in size was attributed to the transition from a predominantly acrylamide backbone (**A2**) to an acrylate backbone (**C1**), further showcasing the role of hydrogen bonding in complexation of pDNA into small nanoparticles since the cationic moieties were comparable. Furthermore, **A1** (73 nm) and **A2** (64 nm) were slightly smaller than their counterparts, **B1** (91 nm) and **B2** (92 nm), which aligned with the EBA and HRA observations. Lastly, a morphology investigation of **C1** polyplexes by Cryo-TEM revealed a spherical morphology (Figure 2E).

### Cytotoxicity and Polymer–Membrane Interaction

**Figure 3:**
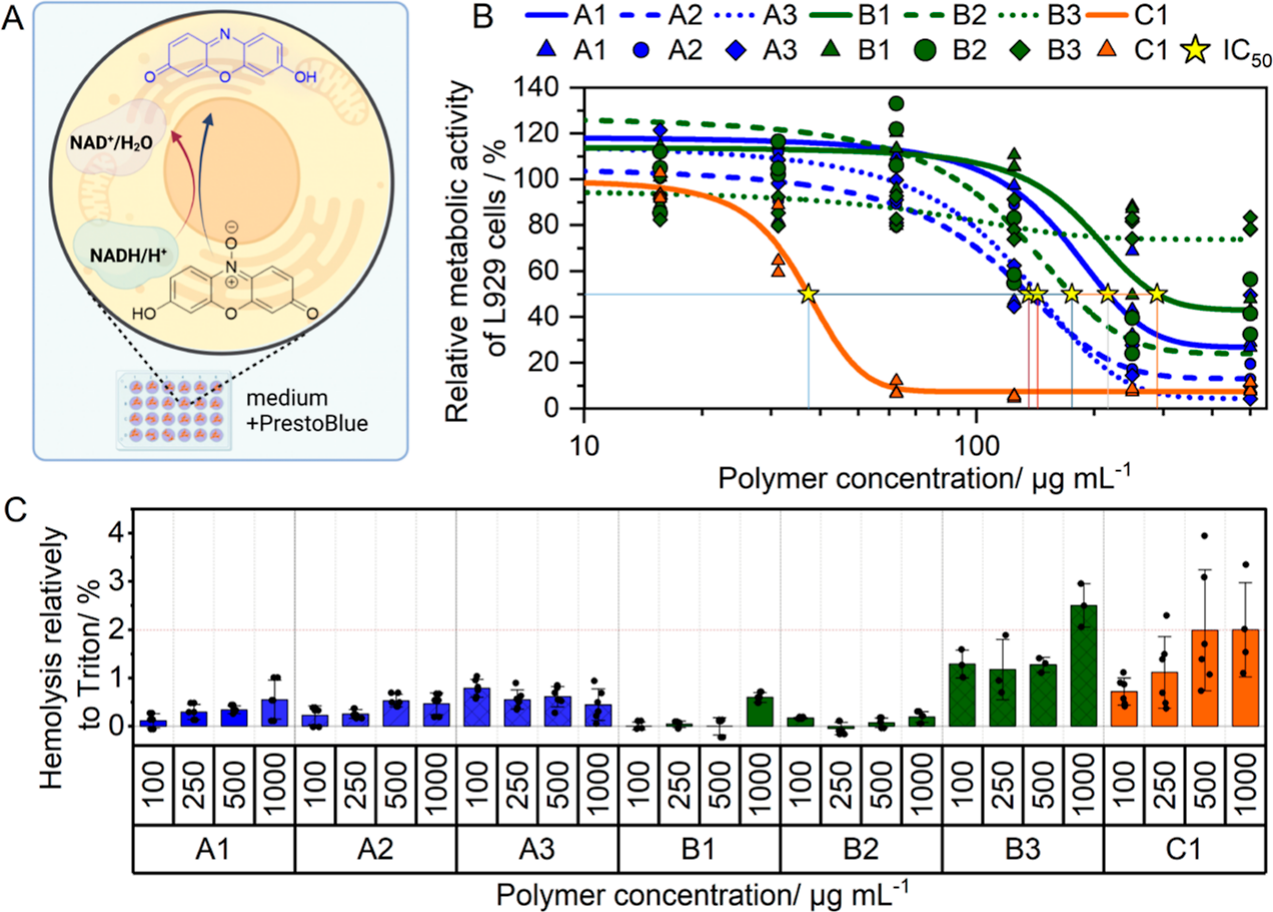
(A) Mechanism of the cytotoxicity assay (PrestoBlue assay). (B) PrestoBlue assay in L929 over 24 h in a full growth medium (D10H). Dots represent values of single repetitions. Lines were fitted, and IC_50_ values were calculated with dose–response function (*n* = 3). Stars indicate the polymer concentration (μg mL^–1^), which induces 50% cytotoxicity. Viability below 70% was considered cytotoxic. (C) Hemolysis assay was performed in triplicate with three different donors. A relative hemolysis of > 2% is considered slightly hemolytic, and > 5% is considered hemolytic.

Biocompatibility is an essential prerequisite for the success of a gene carrier. Polymeric carriers often encounter the toxicity-efficiency dilemma, in which the increase in transfection performance is associated with an increase in cytotoxicity and vice versa.^54^ Polycations can interact with cell surfaces and cause membrane destabilization and, consequently, necrosis.^28,29,55^ However, as cell surface interactions play a pivotal role in enhancing transfection, it becomes imperative to strike a delicate balance between the compatibility of particles with the biological system and their interaction with the cell membrane. Therefore, to assess the cytocompatibility of the polymers, a PrestoBlue assay was conducted in accordance with ISO10993-5.^40^ The assay is based on a fluorometric method and is used to determine the cell’s metabolic activity (Figure 3A). Viability below 70% is considered cytotoxic.^40^

As shown in Figure 3B, **A** and **B** polymer sets with an AAEN side chain exhibit high cytocompatibility. Generally, when comparing the **A** and **B** polymer sets, two distinct observations emerge: an increase in AAEN leads to improved biocompatibility, and substituting DMAEAm with DMAEA also contributes to enhanced biocompatibility as indicated by high IC_50_ and IC_70_ values (Supporting Information; half-maximal inhibitory concentrations are provided in Table S4).

The effect of the latter, *i.e.*, substituting DMAEAm with DMAEA, was particularly evident when extreme molar ratios were analyzed, *i.e.*, P(AAEN_148_-*co*-DMAEAm_50_) (**A1**) *vs* P(AAEN_152_-*co*-DMAEA_50_) (**B1**) and P(AAEN_48_-*co*-DMAEAm_142_) (**A3**) *vs* P(AAEN_49_-*co*-DMAEA_149_) (**B3**), whereby the **B** polymers were less toxic. Moreover, **B3** was nontoxic across the tested concentration range. The improved cytotoxicity profiles of DMAEA containing copolymers are due to the self-hydrolysis nature of DMAEA, which leads to nontoxic byproducts.^27^ In contrast, the increase in cytotoxicity observed for the **A** polymer set is positively correlated with the molar ratio of DMAEAm. While DMAEAm is essential for binding with pDNA due to its cationic charged amine, it has a strong interaction with the negatively charged cellular membrane, leading to decreased cytocompatibility.^28^ Remarkably, an increase in the relative metabolic activity of **A** and **B** polymers, surpassing 100%, was also observed at lower concentrations. This phenomenon can be attributed to AAEN, as its homopolymer nanoparticles demonstrated a similar enhancement of cellular metabolism.^39^ Additionally, this effect was similarly more pronounced with AAEN compared to AEN. Surprisingly, **C1** was notably cytotoxic. This was unexpected, considering that in our previous work, PAEN and PAAEN were found to be nontoxic up to 300 μg mL^–1^.^39^ Therefore, the cytotoxicity profile of **C1** was anticipated to mirror that of **B2** considering their similar molar ratios, with the main difference being the substitution of AEN with AAEN. However, the observed higher cytotoxicity might be linked to the decreased particle stability and poor polymer solubility due to increased hydrophobicity, potentially resulting in increased aggregation at higher concentrations.

To assess the interaction between the polymers and the cellular membrane, isolated erythrocytes were exposed to varying concentrations of polymer in a hemolysis assay. A membrane disruption leads to release of hemoglobin and an increase of the measurable absorbance of the samples. Remarkably, as shown in Figure 3C, both the **A** and **B** polymer sets exhibit less than 2% hemolytic activity. While **C1** also falls within this range for most concentrations, it slightly exceeds the 2% limit only at the highest tested concentration. These observations not only indicate hemocompatibility but also underscore the high cytocompatibility of the polymers.

### Particle Uptake Study

**Figure 4:**
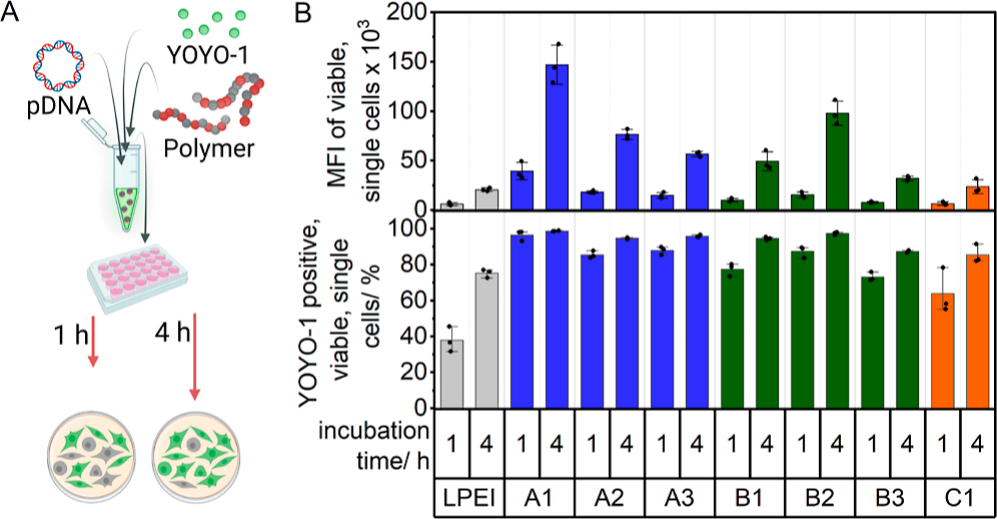
(A) Schematic illustration of complexation between the polymers and pDNA and YOYO-1. The experiment was used to determine the particle uptake behavior. (B) Particle uptake was performed in HEK293T cells with full growth medium (D10H) over 1 and 4 h at N*/P ratio 20 and c(pDNA) = 3 μg mL^–1^ on cells (*n* = 3). The gating strategy can be found in the Supporting Information, Figure S9.

The internalization of the polyplexes with nucleic acids is the first cellular barrier for efficient gene delivery. To determine the uptake efficiency, HEK293T cells are incubated with polyplexes of the EGFP-noncoded plasmid pKMyc which is labeled with the green fluorescent, dimeric cyanine nucleic acid stain YOYO-1. The particle uptake study was performed over 1 and 4 h (Figure 4A).

All polymers demonstrated a fast internalization within the first hour, especially for the **A** polymer series, in terms of YOYO-1 positive cells (Figure 4B). However, when considering the mean fluorescence intensity (MFI), the uptake efficiency for all polymers is time-dependent. Remarkably, **A1** with the highest AAEN content in the **A** polymer set exhibited superior performance out of all of the investigated polymers, followed by **B2**. This outcome highlights that the internalization of the polyplexes is influenced not only by efficient binding but also by an optimized ratio of the monomers within the polymer composition.

### Transfection Efficiency

**Figure 5:**
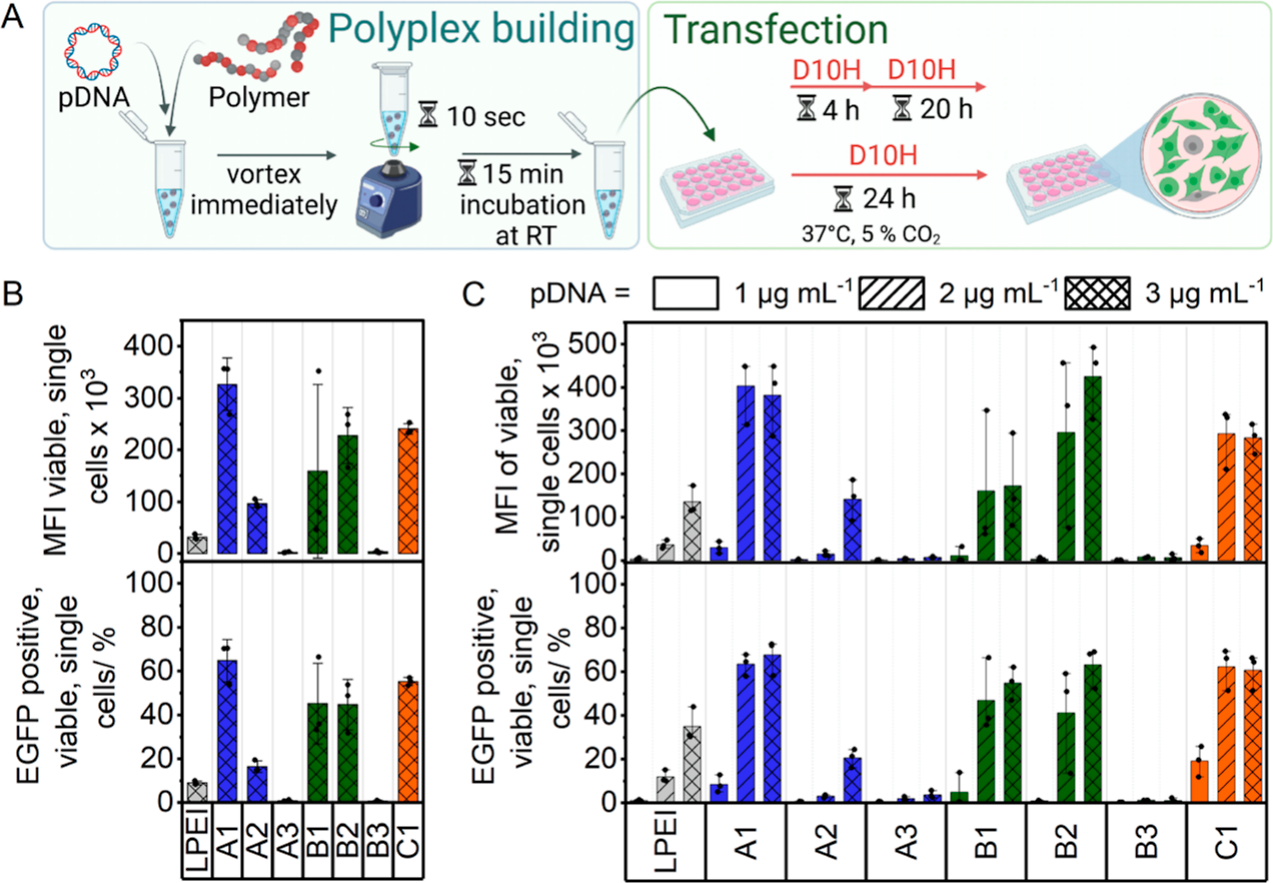
(A) Schematic illustration of the experimental design for determining transfection efficiency under two different conditions (4 + 20 and 24 h). (B) Transfection efficiency was performed in HEK293T cells with full growth medium (D10H) over 4 + 20 h at N*/P 20 and c(pDNA) = 3 μg mL^–1^ on cells (*n* = 3). (C) Performed over 24 h at N*/P 20 and three different concentrations of the genetic material (*n* = 3). Details of statistical tests can be found in the Supporting Information, Tables S6–S11.

The transfection efficiencies of the polymers were evaluated by using HEK293T cells over two different incubation periods at N*/P 20 (Figure 5A). The assessment is based on quantifying the proportion of viable single cells expressing enhanced green fluorescent protein (EGFP-pos. viable single cells) and their MFI (Supporting Information; the gating strategy is shown in Figure S10). Initially, to ascertain the effect of shorter exposure time on the transfection efficiency, the cells were incubated with polyplexes for 4 h at pDNA concentration of 3 μg mL^–1^ in full growth medium (D10H), followed by a medium change to D10H, and further incubated for 20 h (4 + 20 h). Figure 5B illustrates that all polymers, except P(AAEN_48_-*co*-DMAEAm_142_) **A3** and P(AAEN_49_-*co*-DMAEA_149_) **B3**, displayed a transfection performance. However, **A3** exhibited notably low performance, comparable to LPEI, with less than 20% of EGFP-positive single cells, while the rest exhibited transfection efficiencies of ≥40% (Supporting Information; Table S7 shows that **A1**, **B1**, **B2**, and **C1** reveal significantly higher EGFP positive cells). Interestingly, assessment of endosomal release after 4 h using nonpermeable dye calcein revealed low levels of endosomal release for all polymers (Supporting Information; endosomal images are provided in Figures S11 and S12). When the high uptake efficacy of the polymers is considered (Figure 4), it can be concluded that the observed differences in transfection performances were likely due to the slow and varied endosomal release profiles of the polymers.

Afterward, each polymer was tested at three pDNA concentrations (3, 2, and 1 μg mL^–1^) with an extended exposure period of 24 h. As shown in Figure 5C, at a pDNA concentration of 3 μg mL^–1^, a trend similar to that observed at 4 + 20 h was noted, with a gradual increase in the proportion of EGFP-positive single cells and a substantial increase in their corresponding MFI values. The similarity in performance trends at 4 + 20 and 24 h, combined with a substantial increase in MFI values, further supports that the variations in performance are largely due to differences in endosomal release, as long exposure times result in improved release, albeit increased uptake.

Nonetheless, at both tested conditions (4 + 20 and 24 h), **A1**, **B1**, and **C1** were significantly superior to the commonly used transfection agent control, LPEI, at pDNA ≥ 2 μg mL^–1^ and **B2** at pDNA ≥ 3 μg mL^–1^ in terms of EGFP-positive cells (Supporting Information; calculated statistics of EGFP-positive cells are shown in Table S9). Additionally, **A1** and **B2** were significantly superior to LPEI in terms of MFI at pDNA ≥ 3 μg mL^–1^ (Supporting Information; calculated statistics of MFI are shown in Table S11). Furthermore, it can be observed from the transfection performance of each polymer set that an increase in the molar ratio of AAEN, hence niacin, leads to an enhancement in transfection performance. This pattern is similar to the cytotoxicity profile observed in Figure 3B. Moreover, CytoTox-ONE assay results revealed that all polymers exhibit low membrane destabilizing effects (Supporting Information; CytoTox-ONE plots are shown in Figure S13). This finding is aligned with the hemolysis results (Figure 3C). Overall, this emphasizes the vital role of the niacin-derived monomer in augmenting the transfection performance and biocompatibility.

When comparing P(AAEN_148_-*co*-DMAEAm_50_) (**A1**) and P(AAEN_123_-*co*-DMAEAm_75_) (**A2**), the latter only shows low performance at the highest pDNA concentration (3 μg mL^–1^), while **A1** maintains relatively high transfection efficiency at pDNA concentrations ≥ 2 μg mL^–1^ and experiences a drastic decrease of performance at the lowest pDNA concentration (1 μg mL^–1^). As such, the performance of the **A** polymer set improved in the order of **A1** > **A2** > **A3**. On the other hand, a different trend is observed for P(AAEN_152_-*co*-DMAEA_50_) (**B1**) *vs* P(AAEN_120_-*co*-DMAEA_72_) (**B2**), where both polymers maintain enhanced and comparable proportions of EGFP-positive cells at pDNA concentrations of 2 and 3 μg mL^–1^. Interestingly, **B2** outperformed **B1** in terms of MFI values. This aligns with the uptake observations (Figure 4B), where **B2** shows a higher MFI than **B1**, implying that both endosomal release profiles and uptake profiles contribute to the observed performance differences. Consequently, the effectiveness in performance of the **B** polymers was concluded to be in the order of **B2** ≈ **B1** > **B3**.

Analyzing transfection performance of the polymers with similar molar ratios but different compositions, *i.e.*, P(AAEN_123_-*co*-DMAEAm_75_) (**A2**), P(AAEN_120_-*co*-DMAEA_72_) (**B2**), and P(AEN_115_-*co*-DMAEA_65_) (**C1**), reveals intriguing insights. At pDNA concentrations of 2 and 3 μg mL^–1^, the transfection performance in terms of EGFP-positive cells and their corresponding MFI values follows the order **B2** > **C1** > **A2**. The results of **B2** and **C1** indicate that substituting AAEN with AEN has a minimal impact on transfection performance. Interestingly, **C1** surpasses **B2** and even **A1** at the lowest pDNA concentration (Supporting Information; a closer look at the transfection results at 1 μg mL^–1^ is shown in Figure S14). Considering the EBA and HRA results (Figure 2B,C), the polymers’ ability to bind and release pDNA probably plays a role in the observed results. **C1** demonstrates superior binding compared to **B1** and **B2**, while exhibiting faster release profiles. This implies that at lower pDNA concentrations and thus lower polymer concentrations, **B1** and **B2** may be prone to genetic material loss, potentially explaining performance differences. Conversely, the strong binding propensity of the **A** polymers potentially hinders pDNA release (Figure 2C). This underscores the importance of striking a balance between binding to shield genetic material from serum interactions and facilitating its release for transfection.

**Figure 6:**
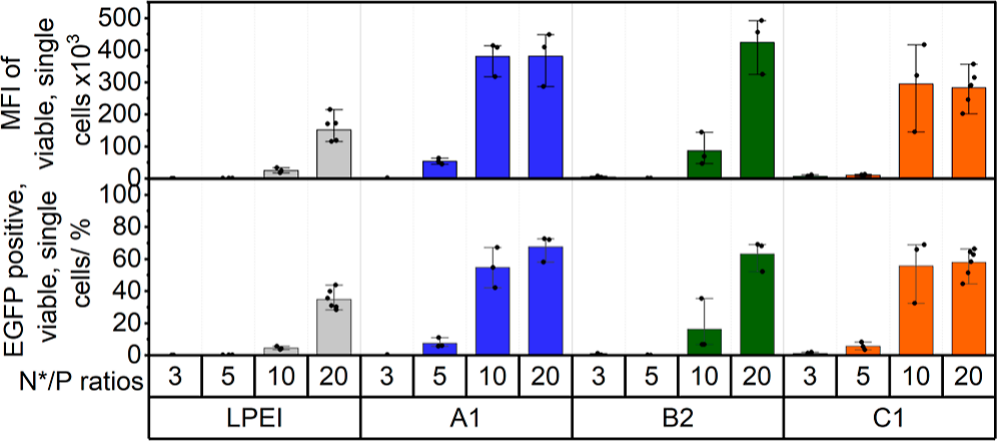
Transfection efficiency was performed in HEK293T cells with full growth medium (D10H) over 24 h at different N*/P ratios and c(pDNA) = 3 μg mL^–1^ on cells for **A1**, **B2**, and **C1** (*n* ≥ 3). Details of statistical tests can be found in the Supporting Information, Tables S12–S15.

To gain a deeper understanding of how N*/P ratios affect performance, additional transfection efficiency tests were conducted at N*/P ratios of 3, 5, and 10 for **A1**, **B2**, and **C1**. Figure 6 reveals a gradual improvement in transfection efficiency with an increased N*/P ratio for all polymers. However, at N*/P 10, both **A1** and **C1** achieve transfection efficiencies comparable to those at N*/P 20. This outcome showcases the superior performance of **A1** and **C1**, which allows for optimal performance at low material input. This is particularly useful since the use of excess polymer is minimized. Furthermore, the CytoTox-ONE assay again revealed low membrane destabilizing effects across all tested N*/P ratios (Supporting Information; CytoTox-ONE plots at different N*/P ratios are shown in Figure S15).

### Immunomodulatory Role of Niacin-Derived Copolymer

**Figure 7:**
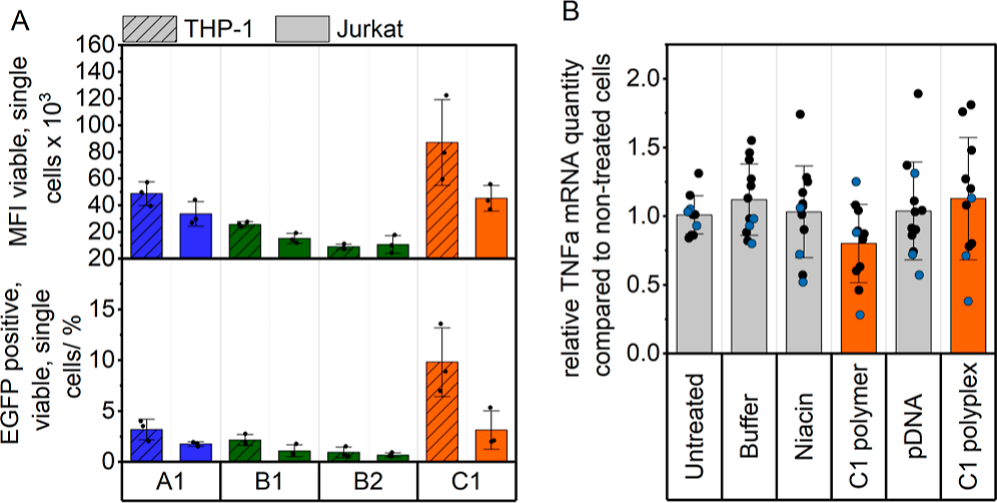
(A) Transfection efficiency was performed with **A1**, **B1**, **B2**, and **C1** in hard-to-transfect suspension cell lines THP-1 and Jurkat. Transfection was performed in full growth medium (R10H) over 24 h at N*/P 20 and 3 μg mL^–1^ genetic material on cells (*n* = 3). (B) Fold change (compared to non-treated cells) of the relative *TNF-α* mRNA expression (normalized to *GAPDH*) of *ex vivo* murine classical monocytes from the bone marrow. One point represents one biological replicate (mice, blue = male, black = female). Mean with standard deviation is depicted. Details of statistical tests can be found in the Supporting Information, Tables S16 and S17.

First, transfection performance of the top-performing polymers (**A1**, **B1**, **B2** and **C1**) was evaluated using immortalized THP-1 and Jurkat cells. The transfection investigation was performed in full growth medium (R10H) over 24 h at N*/P 20 and a pDNA concentration of 3 μg mL^–1^. THP-1, a monocytic leukemia cell line, serves as a widely recognized model for studying human monocytes and macrophages, while Jurkat T cells, a T lymphocyte line, are employed in research on acute T cell leukemia and T cell signaling.^56−58^ Though transfection efficacy in both cell lines is advantageous for treating immune-related disorders, they are both difficult to transfect.^59,60^ As shown in Figure 7A, the polymers demonstrated lower performance compared to HEK293T cells (Figure 5C). However, given the inherent difficulties in transfecting these cell lines, the performance is positive. Notably, P(AEN_115_-*co*-DMAEA_65_) (**C1**) outperformed P(AAEN_148_-*co*-DMAEAm_50_) (**A1**), P(AAEN_152_-*co*-DMAEA_50_) (**B1**), and P(AAEN_120_-*co*-DMAEA_72_) (**B2**) in both Jurkat and THP-1 cells. Regarding the latter, **C1** exhibited approximately a 3-fold improvement compared to the second-best, **A1**, for both EGFP-positive cells and MFI values.

Additionally, the cytocompatibility of the polymers to the THP-1 and Jurkat cell lines remained high at the polymer concentrations used for the transfection (N*/P 20, c(pDNA) = 3 μg mL^–1^ on cells) over AAEN, emphasizing the potential of niacin-derived components to target monocytes. To this end, when considering future applications, it is important to consider the immunomodulation properties of the used materials. In addition, niacin is known to have anti-inflammatory effects (such as a decreased secretion of the pro-inflammatory cytokine *TNF-α*) and is holding promising results as a therapeutic reagent.^61−64^ To test whether the niacin-based polymer, **C1**, might have anti-inflammatory effects similar to those of niacin, murine classical monocytes were isolated from the bone marrow and treated in culture with the following: NaOAc buffer (contained as solvent in all other groups as well), niacin, polymer **C1** pure pDNA, and the polyplex (pDNA + **C1**) for 24 h. Simultaneously, untreated cells were used as a control. After 18 h, cells were checked under a light microscope to observe the morphology. No condition induced obvious morphological changes or excessive amounts of dead cells (not depicted). After the treatment, a possible immunomodulatory response of the monocytes *via TNF-*α mRNA expression of male and female mice was analyzed (Figure 7B), and no significant differences were found. However, cells treated with the **C1** polymer have the lowest mean of all groups with 0.80 compared to buffer (1.12), niacin (1.03), pDNA (1.04), and the polyplex (1.13) (Supporting Information; descriptive statistics are provided in Table S17). Since TNF-α serves as a endocrine and paracrine mediator, influencing inflammatory and immune responses,^65,66^**C1** exhibits promise in avoiding inflammatory responses, a desirable trait for gene carriers. Even though pure niacin is reported to be anti-inflammatory,^61−64^ an effect which could not be reproduced in this study, changes might be more pronounced on the protein level or under acute inflammatory stimuli. It would be beneficial to counteract this in future studies. However, in summary, a significant increase of *TNF-*α expression was not proved in any kind of treatment, which could be useful for potential novel gene delivery concepts, where no simultaneous immune activation is envisaged, *e.g.,* diseases beyond vaccination, such as clinical patients with acute inflammatory diseases. These findings underscore the important potential of the niacin-derived copolymer (a combination of AEN and DMAEA) as a safe gene carrier.

## Conclusions

A library of cationic hydrophobic copolymers (**A1–A3**, **B1**–**B3**, and **C1**), incorporating niacin-derived monomers (AAEN or AEN) in combination with either DMAEAm or DMAEA, was successfully synthesized by RAFT polymerization. The technique yielded polymers with narrow monomodal mass distributions (dispersities of 1.3, except for **C1**, which had a value of 1.4). Titration studies on the polymers revealed apparent p*K*_a_ values of DMAEA and DMAEAm ranging from 7.2 to 8.0.

Regarding the biological investigations, an increase in AAEN in both polymer sets resulted in improved transfection and biocompatibility profiles. However, when the cytotoxicity associated with DMAEA and DMAEAm is considered, it is evident that DMAEA-containing polymers offer the optimal balance between transfection efficiency and cytotoxicity. The low cytotoxicity of DMAEA is due to its self-hydrolysis nature, yielding low toxic byproducts. Alternatively, a combination of AAEN and low amounts of DMAEAm also achieves an optimal compromise between these two factors as showcased by **A1**. Overall, the exceptional performance of the top performers (**A1**, **B1**, **B2**, and **C1**) in HEK293T cells addresses several pivotal challenges in gene delivery, including (i) affinity to complex genetic material and formation of suitable polyplexes, (ii) delivering pDNA under full serum conditions, highlighting particle stability, (iii) maintaining high transfection and biocompatibility levels across varying pDNA concentrations and N*/P ratios, (iv) showing promise in delivery to different cell lines, and (v) nonimmunoactivity property.

Finally, when the transfection performance of the best performers on difficult-to-transfect cells (THP-1 and Jurkat cells) was evaluated, **C1** emerged as the top performer, especially in THP-1 cells, followed by **A1**, **B1**, and **B2**, respectively. Importantly, **C1** demonstrated biocompatibility with the cells, as did the other polymers. Remarkably, *ex vivo* investigations of **C1** on its effect on *TNF-*α mRNA levels showed that it did not significantly increase the levels of *TNF-*α, even showing a slight decrease. This finding could potentially be useful for transfection experiments with the polyplex, as an increased immune response might not be feasible in all situations, such as in clinical patients with acute inflammatory diseases. However, further experiments should be conducted to determine whether the polymer **C1** totally exhibits a potential in avoiding inflammatory responses. Overall, the performance of the niacin-derived polymers showcases the potential of utilizing nutrient-derived polymers to unlock the performance of gene carriers.

## Supporting Information Available

Instruments and materials, monomer and polymer synthesis and characterization, polymerization kinetics procedures, titrations, degradation of DMAEA, N*/P ratio calculations, EBA and HRA, cytocompatibility (PrestoBlue and CytoTox-One assay), particle uptake study, transfection efficiency, endosomal escape, antibody mix for murine monocyte staining, and statistics (PDF)
