# Supplementary material for: Optimizing Biocompatibility and Gene Delivery with DMAEA and DMAEAm: A Niacin-Derived Copolymer Approach
Source: Biomacromolecules. 2024 Jul 4;25(8):4749–61. doi: 10.1021/acs.biomac.4c00007 (PMC11323007; doi:10.1021/acs.biomac.4c00007)
Supplement: Supplementary file 1 — bm4c00007_si_001.pdf [file bm4c00007_si_001.pdf]

Supporting Information:

Optimizing Biocompatibility and Gene Delivery with DMAEA and DMAEAm: A Niacin-Derived Copolymer Approach

Prosper P. Mapfumo, [a‡] *Liên S. Reichel,* [a‡] *Thomas André,*[c] *Stephanie Hoeppener,* [a,b] *Lenhard K. Rudolph,*[c] *Anja Traeger*[a, b]*

[a] Institute of of Organic and Macromolecular Chemistry (IOMC), Friedrich Schiller University Jena, Humboldtstrasse 10, 07743 Jena, Germany

[b] Jena Center for Soft Matter (JCSM), Friedrich Schiller University Jena, Philosophenweg 7, 07743 Jena, Germany

[c] Leibniz Institute on Aging-Fritz Lipmann Institute, 07745 Jena, Germany

Instruments

Nuclear magnetic resonance (NMR) spectroscopy. ^{1}H NMR (300 MHz) and DEPT ^{13}C (75 MHz) spectra were recorded on a Bruker AC 300 MHz spectrometer at 300 K. The delay time (d1) was set at 1 s for ^{1}H NMR and 2 s for DEPT ^{13}C. Chemical shifts (δ) are reported in ppm.

Size exclusion chromatography (SEC). Dimethylacetamide (DMAc)-SEC was conducted using an Agilent 1200 series instrument equipped with differential refractive index (DRI) and UV/vis (DAD) detector. The liquid chromatography system used 1 × PSS GRAM 30 Å column (300 × 0.8 mm, 10 µm particle size) and 1 × PSS GRAM 1000 Å column (300 × 0.8 mm, 10 µm particle size). The DMAc eluent contained 0.21 wt.% LiCl as additive. Samples were run at 1 mL min^{-1} at 40 °C. Analyte samples were filtered through a polytetrafluoroethylene (PTFE) membrane with 0.45 µm pore size prior to injection. Poly(methyl methacrylate) (PMMA) narrow standards (PSS) were used to calibrate the SEC system. Experimental $M_{n,SEC}$ and $Đ$ (M_w/M_n) values of synthesized polymers were determined using PSS WinGPC UniChrom GPC software.

Differential scanning calorimetry (DSC). Measurements were performed on a Netzsch DSC 204 F1 Phoenix under N_2 atmosphere from −100 to 160, −100 to 150, −190 to 100, and −190 to 150 °C. For every measurement, three heating runs were recorded. The first and second runs were performed at a heating rate of 20 K min^{-1}, and the third one at 10 K min^{-1}. The cooling rates between the first and second runs and between the second and third runs were set at 20 and 10 K min^{-1}, respectively. Glass-transition temperatures (T_g, inflection values) were determined from the second heating run. Thermograms were analyzed using the Netzsch Proteus Thermal Analysis 8.0.2 software applying the smoothing option if needed to analyze the T_g value.

Dynamic Light Scattering (DLS). The hydrodynamic diameters of the nano assemblies were monitored by (DLS) using a Zetasizer Nano ZS (Malvern Instruments, Germany) with a He−Ne laser operating at a wavelength of 633 nm.

Flow cytometry. Flow cytometry was conducted on CytoFLEX Beckmann Coulter, Brea, CA, U.S. For each experiment, 20^4 cells per sample were analyzed using bandpass filter 510 ± 10 nm with signal attenuation (OD1).

Multi-plate reader. PrestoBlue™ assay, CytoTox-ONE™ assay, ethidium bromide binding assay, heparin dissociation assay and hemolysis assay were measured with the multi-plate reader Tecan infinite M200Pro, Germany, using settings described in the respective method section.

Fluidlab R-300 anvajo: was used to count cells for cell seeding procedures.

Centrifuge 5804R: Eppendorf, Wesseling-Berzdorf, Germany.

Confocal laser scanning microscope (CLSM) was used to characterize the endosomal release. LSM880, Elyra PS.1 system (Zeiss, Germany), a magnification of 40x at 37 °C, and a 40 × 1.4 NA plan apochromat oil objective, argon laser with λEx 488 nm (0.3%) and 405 nm (0.5%), emission signal was detected in the range of 410-469 nm (Hoechst) and 490-544 nm (Calcein) with a digital gain of 1 and with a master gain of 750 and a pinhole of 27 µm were used. Non digital offset was used. Images were acquired using the ZEN software, version 2.3 SP1 (Zeiss, Germany). To avoid cross talk between the different channels, Hoechst 33342 and calcein were imaged consecutively in different tracks.

Materials

2-(dimethylamino)ethyl acrylate (DMAEA), 2-Hydroxyethyl acrylate (HEA) (96 %), *N*-Hydroxyethyl acrylamide (HEAm) (97 %), Nicotinoyl chloride hydrochloride (97 %), Triethylamine (TEA) (for synthesis), Acryloyl chloride (97 %), Hydrogen chloride solution (HCl) (4 M in dioxane) and 4,4′-Azobis(4-cyanovaleric acid) (ACVA) (98%) were obtained and used as received from Sigma-Aldrich. 2,2'-Azobis(2,4-dimethylvaleronitrile) (V-65B) and *N*-[2-(dimethylamino)ethyl]acrylamide (DMAEAm) were obtained from FUJIFILM Wako Chemicals and TCI chemicals respectively. (propionic acid)yl butyl trithiocarbonate (PABTC) was prepared following a previously reported procedure.[1] Tetrahydrofuran (THF), dichloromethane (DCM), ethyl acetate (EtOAc), diethyl ether, hexane, dioxane and methanol (MeOH) were distilled on site. DMAEA and DMAEAm were filtered through a small basic alumina column plug before use.

For biological investigations all the following materials were ordered from the suppliers stated in brackets: TC treated cell culture flasks and 96-U-bottom well plate (Greiner Bio-One International GmbH and Labsolute, Th. Geyer GmbH & Co. KG), TC treated multi-well cell culture plates (VWR International GmbH), black 96-well plates (Nunc, Thermo Fisher), disposable folded capillary cells (DTS1070, Malvern Instruments, Herrenberg, Germany). L929 cells,THP-1 cells and Jurkat were purchased from CLS Eppelheim, Germany, HEK293T (DSMZ, Braunschweig, Germany), Dulbecco's modified eagle medium (DMEM), RPMI 1640 with Stable Glutamine (RPMI) and 4-(2-hydroxyethyl)-1-piperazineethanesulfonic acid) (HEPES) buffer 1M, phosphate-buffered saline (PBS), fetal bovine serum (FCS), Trypsin-EDTA and Penicillin-Streptomycin were purchased from Capricorn Scientific, Opti-MEM™ reduced serum medium and PrestoBlue™ cell viability reagent (Thermo Fisher Scientific), CytoTox-ONE™ Homogeneous for membrane integrity assay (Promega), 1% ethidiumbromide solution, agarose-HR PLUS (Carl Roth, Karlsruhe, Germany), green gel loading buffer (Jena Biosciences, Jena, Germany), heparin sodium salt (Alfa Aesar, Haverhill). pDNA encoding the enhanced green fluorescent protein (EGFP) for transfection studies was isolated with the Giga Plasmid Kit (Qiagen, Germany) from E. coli containing pEGFP-N1 (4.7 kb, Clontech, USA), pKMyc was a gift from Ian Macara (Addgene plasmid #19400;http://n2t.net/addgene: 19400;RRID: Addgene_19400). Addgene_19400), Hoechst (Invitrogen), Calcein and Hanks'5 balanced salt solution (Sigma Aldrich), 8 well chamber slide (ibidi GmbH), nicotinic acid (Sigma-Aldrich). The following reagents were used to isolate and stain for classical Monocytes from murine bone marrow: Ly-6C Antibody, Anti-mouse, APC, REAfinity™, Clone REA796, Order No. 130-111-779, Milteny Anti-Mo CD115 (c-fms), eBioscienceTM, PE, Clone: AFS98, Invitrogen, 12-1152-82 APC/Cyanine7, anti-mouse/human CD11b, clone: M1/70, Isotype: Rat IgG2b, k, Biolegend, Cat. 101226 FcR Blocking Reagent mouse, Milteny, Order no.: 130-092-575 Anti-APC MicroBeads, Milteny, Order no.: 130-090-855. After obtaining a pure population of classical Monocytes, the following medium was added to the cells for incubation (prepared following Haag and Murthy2): DMEM high glucose media (Sigma, catalog number: D6429-500ML), 10% FBS (Sigma, catalog number: F7524), 1× GlutaMAX (Gibco, catalog number: 35050061), 1× Penicillin/Streptomycin (Gibco, catalog number: 10378-016), 5 ng/ml rmGM-CSF (R&D

Systems, catalog number: 415-ML010), 2.5 ng/ml rmIL-4 (R&D Systems, catalog number: 404-ML-010).

Methods

Monomer Synthesis and Characterization

*2-(acryloyloxy)ethyl nicotinate **(AEN)***. Adopted from previously reported procedure.[3]

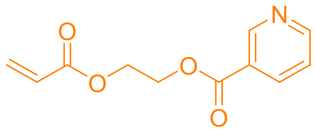

Nicotinoyl chloride hydrochloride (12.88 g, 72.88 × 10^{-3} moles) was suspended in DCM (150 mL) and cooled with an ice bath while stirring. Using a dropping funnel, a dissolved mixture of HEA (6 g, 51.67 × 10^{-3} moles) and TEA in DCM (50 mL), was added dropwise to the suspended solution. After, the reaction was left on ice for 30 min and then left at room temperature (RT) overnight. For workup, the reaction was quenched with water (50 mL), transferred to extraction funnel and additional water (150 mL) was added. The organic layer was further washed with water (3 × 250 mL), and $NaHCO_2$ (2 × 200 mL), dried with $MgSO_4$ and filtered. The crude product was concentrated and purified on a silica plug using diethyl ether (200 mL fractions). The fractions containing the product were isolated and dried under reduced pressure at 35 ^{o}C to yield a yellow oil (75%).

2-acrylamidoethyl nicotinate **(AAEN)**. Adopted from previously reported procedure.[3]

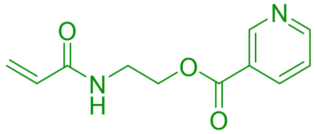

Nicotinoyl chloride hydrochloride (13.00 g, 72.96 × 10^{-3} moles) was suspended in DCM (200 mL) and cooled using an ice bath while stirring. Using a dropping funnel, a dissolved mixture of HEAm (6.0 g, 52.11 × 10^{-3} moles) and TEA (21.1 mL, 151.13 × 10^{-3} moles) in DCM (50 mL), was added dropwise to the suspended solution. The reaction was left on ice for 30 min and then left at RT overnight. For workup, DCM was first removed under reduced pressure, and the crude product was redissolved in THF (300 mL) and filtered. The filtered solution was concentrated and dried on celite. The crude product purified using flash column chromatography EtOAc: MeOH (20:1) to yield a yellow solid (68%).

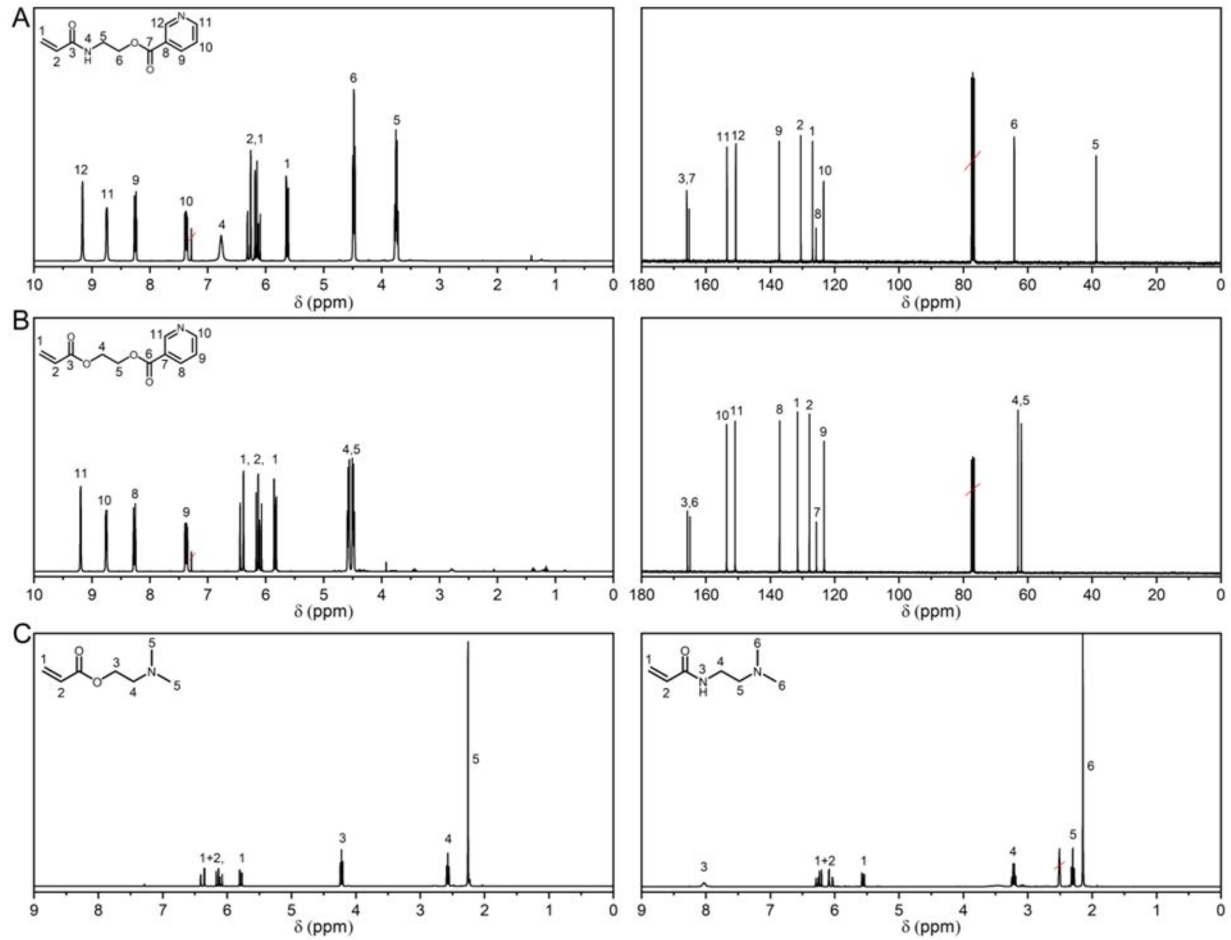

Figure S1. ^{1}H NMR (300 MHz) and ^{13}C NMR (75 MHz) spectra of the synthesized monomers, AAEN (A), AEN (B) and ^{1}H NMR of commercially acquired DMAEA (C, left) and DMAEAm (C, right). DMAEA and DMAEAm were measured using DMSO-d_6 as a solvent while AAEN and AEN were measured using $CDCl_3$.

Polymer Synthesis and Characterization

Calculations for RAFT Polymerization

Monomer conversion (p) was calculated from ^{1}H NMR data by comparing the integrals of vinyl peaks (~5.5-6.2 ppm) against an external reference (1,3,5-trioxane, ~5.1 ppm) before (t = 0) and after (t = final) polymerization. The theoretical number-average molar mass ($M_{n,th}$) then calculated using Equation S1:

$$M_{n,th}\left(\frac{g}{mol}\right) = (Mw_{monomer} * DP * p) + Mw_{PABTC} \quad \text{(Equation S1)}$$

Where DP is the target degree of polymerization of each monomer and, Mw_{monomer}, and Mw_{CTA} are the molecular weight of the monomers, and PABTC, respectively, and p is the monomer conversion of each monomer.

Synthesis of $PDMAEA_{125}$

PABTC (30.0 mg, 1.26 × 10^{-4} moles), DMAEA (2.3 g, 1.64 × 10^{-2} moles), dioxane (1.7 g), a 0.5 wt.% solution of ACVA in dioxane (489.0 mg, 2.45 mg ACVA, 8.73 × 10^{-6} moles) and 1,3,5-trioxane (external NMR standard, 30 mg) were respectively introduced to a 8 mL microwave vial equipped with a magnetic stirring bar. The vial was sealed, and the solution deoxygenated by bubbling argon through it for 10 min. The vial was placed in an oil bath at 70 °C and allowed to stir for 6.5 h. Samples were taken at the beginning, and at the end (6.5 h) to determine conversion by ^{1}H NMR and molecular weight distribution was monitored by SEC. The polymer was purified by precipitation in cold hexane (stored in - 78 ^{o}C freezer) 3 times, redissolving in THF each time. After the last precipitation, the polymer was redissolved in THF and dried under vacuo.

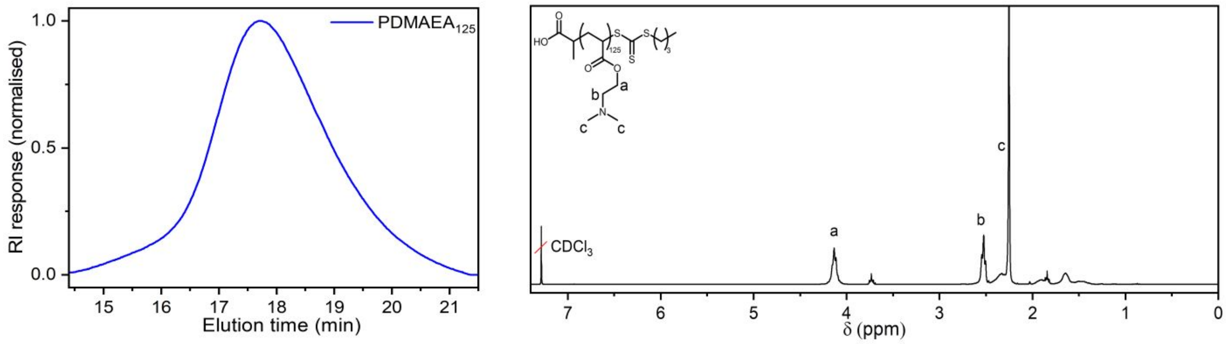

Figure S2. SEC trace using (DMAc + 0.21 % LiCl) as eluent and PS calibration and a ^{1}H NMR of $PDMAEA_{125}$ set using $CDCl_3$ as solvents.

Polymer library synthesis: The polymer synthesis for the library was performed as described in the main text. Table S1 shows the quantities used for the synthesis and Figure S3 shows the characterization of the purified polymers by ^{1}H NMR and SEC. The added quantities for C1 are shown in the main text.

Table S1 Summarized quantities added for the polymerization of **A** and **B** polymer series. Monomer conversions determined by ^{1}H NMR are also shown.

	P(AAEN$_x$-co-DMAEAm$_y$) (A1-A3)			***P(AAEN$_x$-co-DMAEAm$_y$) (B1-B3)***			***P(AEN$_x$-co-DMAEA$_y$) (C1)***
	**A1**	**A2**	**A3**	**B1**	**B2**	**B3**	**C1**
**m_{AAEN} added (mg)**	1.68	1.40	0.57	1.85	1.53	0.63	-
**n_{AAEN} added (moles)**	7.63 × 10^{-3}	6.38 × 10^{-3}	2.60 × 10^{-3}	8.39 × 10^{-3}	6.96 × 10^{-3}	2.85 × 10^{-3}	-
**m_{DMAEAm} added (mg)**	0.37	0.55	1.08	938	940	939	-
**n_{DMAEAm} added (moles)**	2.60 × 10^{-3}	3.86 × 10^{-3}	7.63 × 10^{-3}	4.26 × 10^{-3}	4.27 × 10^{-3}	4.27 × 10^{-3}	-
**m_{DMAEA} added (mg)**	-	-	-	0.41	0.61	1.23	-
**n_{DMAEA} added (moles)**	-	-	-	2.85 × 10^{-3}	4.24 × 10^{-3}	8.60 × 10^{-3}	-
**$Conv_{AAEN}$**	81%	81%	78%	76%	72%	73%	72%
**$Conv_{AEN}$**	-	-	-	-	-	-	-
**$Conv_{DMAEA}$**	-	-	-	73%	72%	72%	72%
**$Conv_{DMAEAm}$**	81%	81%	78%	-	-	-	-

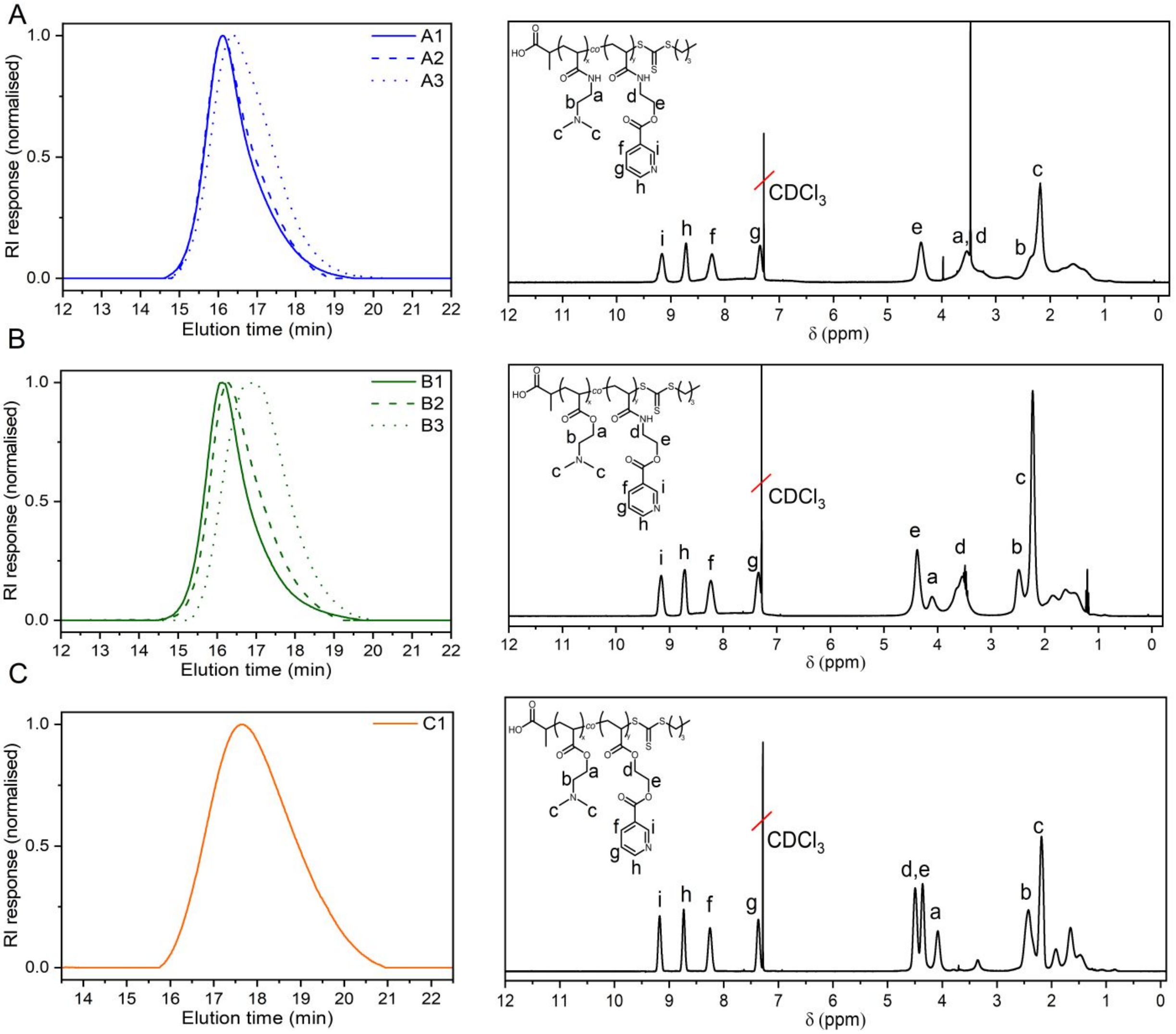

Figure S3. SEC traces using (DMAc + 0.21 % LiCl) as eluent and PMMA calibration and a typical ^{1}H NMR of each polymer set using $CDCl_3$ as solvents.

Polymerization kinetics procedures

P(AAEN$_x$-co-DMAEAm$_y$): PABTC (8.0 mg, 3.36×10^{-5} moles), DMAEAm (0.60 g, 4.19×10^{-3} moles), AAEN (0.92 g, 4.19×10^{-3} moles), Dioxane (6.3 g), a 1 wt.% solution of V-65 in dioxane (0.72 g, 7.22 mg V-65b, 2.80×10^{-5} moles) and 1,3,5-trioxane (external NMR standard, (31 mg) were respectively introduced to a 8 mL microwave vial equipped with a magnetic stirring bar. The vial was sealed, and the solution deoxygenated by bubbling argon through it for 10 min. The vial was placed in an oil bath at 50 °C and allowed to stir for 4 h. Kinetic samples were taken prior to start, then after 1 h, followed by 30 min intervals until 4 h. ^{1}H NMR and SEC were used for monitoring molar mass distributions and monomer conversion (Figure S4A).

P(AAEN$_x$-co-DMAEA$_y$): PABTC (7.5 mg, 3.15×10^{-5} moles), DMAEA (0.57 g, 3.96×10^{-3} moles), AAEN (0.86 g, 3.90×10^{-3} moles), Dioxane (3.7 g), a 0.5 wt.% solution of ACVA in dioxane (331.0 mg, 1.65 mg ACVA, 5.90×10^{-6} moles) and 1,3,5-trioxane (external NMR

standard, (29.3 mg) were introduced to a 8 mL microwave vial equipped with a magnetic stirring bar. The vial was sealed, and the solution deoxygenated by bubbling argon through it for 10 min. After, the vial was placed in an oil bath at 70 °C and allowed to stir for 12 h. Kinetic samples were taken prior to start, then at 1 h intervals for 6 h, followed by 2 h until 12 h. ^{1}H NMR and SEC were used for monitoring molar mass distributions and monomer conversion (Figure S4B).

P(AEN$_x$-co-DMAEA$_y$): PABTC (10.0 mg, 4.19 × 10^{-5} moles), DMAEA (0.37 g, 2.56 × 10^{-2} moles), AEN (0.85 g, 3.86 × 10^{-3} moles), Dioxane (2.9 g), a 0.5 wt.% solution of ACVA in dioxane (0.45 g, 2.25 mg ACVA, 8.02 × 10^{-6} moles) and 1,3,5-trioxane (external NMR standard, (28 mg) were introduced to a 20 mL microwave vial equipped with a magnetic stirring bar. The vial was sealed, and the solution deoxygenated by bubbling argon through it for 10 min. The vial was placed in an oil bath at 70 °C and allowed to stir for 15 h. Kinetic points were taken every 2 h for 12 h and finally after 3 h. ^{1}H NMR and SEC were used for monitoring molar mass distributions and monomer conversion (Figure S4C).

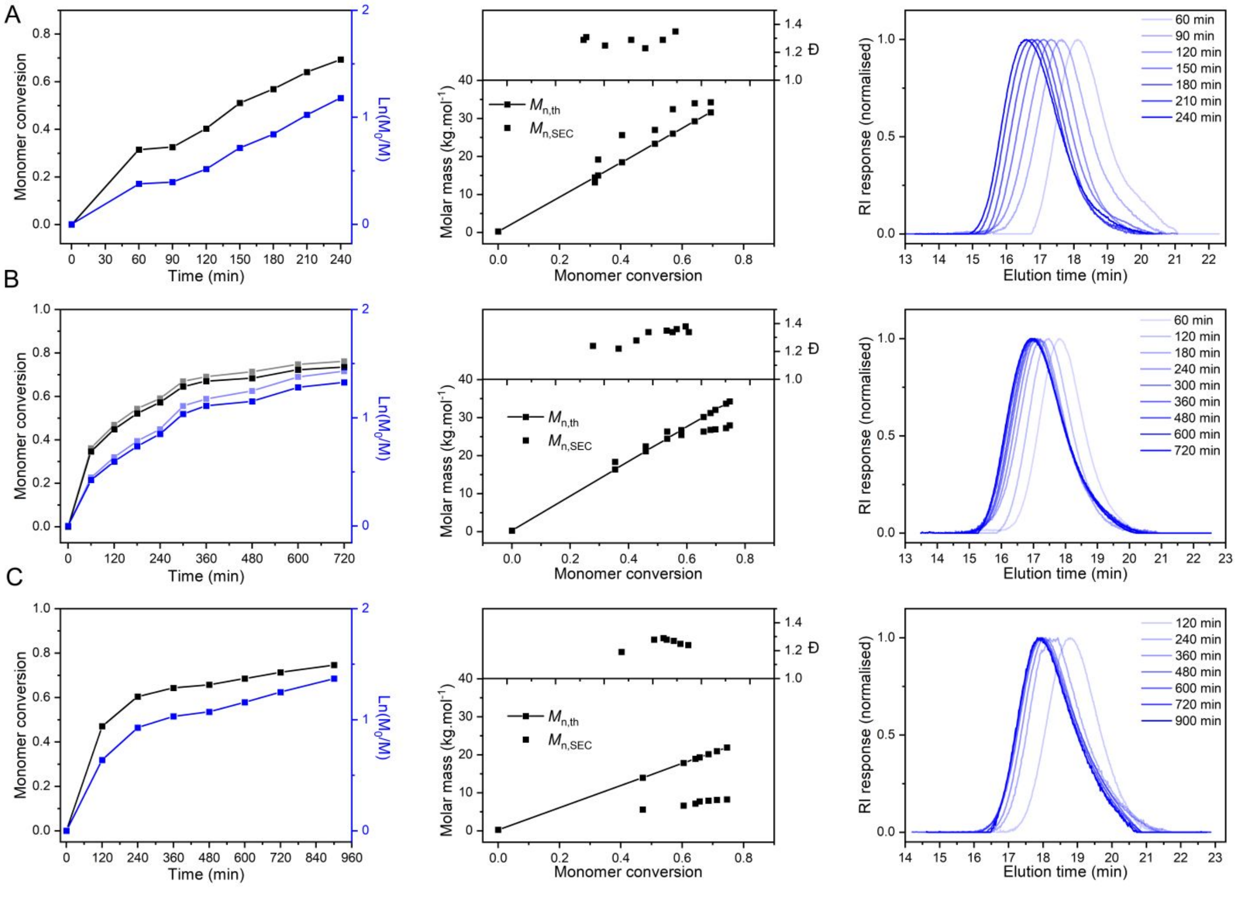

Figure S4. Summary of polymerization kinetics for P(AAEN$_x$-*co*-DMAEAm$_y$) (A), P(AAEN$_x$-*co*-DMAEA$_y$) (B), and P(AEN$_x$-*co*-DMAEA$_y$) (C). Conversion from ^{1}H NMR, molar masses determined by SEC using (DMAc + 0.21 % LiCl) as eluent and PMMA calibration. For each kinetic, the three plots display the following: the left plot illustrates monomer conversion over time, the middle plot shows the comparison of calculated and measured molar masses at different monomer conversions, and the right plot shows SEC traces.

Titrations

The degree of charge (DoC) at different pH values was calculated by Equation S2 as the amount of negatively or positively charged units per total amount of amine groups[4]:

$$R-NH^{+}(CH_2)_2+OH^{-}+H_2O\rightleftharpoons R-N(CH_2)_2+H_3O^{+}+OH^{-}$$

$$\text{DoC} = \frac{[R-NH^{+}(CH_2)_2]}{[R-N(CH_2)_2]_{tot}}\cdot 100 \qquad \text{(Equation S2)}$$

Subsequently, a curve of DoC (y-axis) and pH (x-axis) was plotted. The pK_a values were determined as the pH value where the DoC was 50 % (y = 50) as shown in Figure S5.

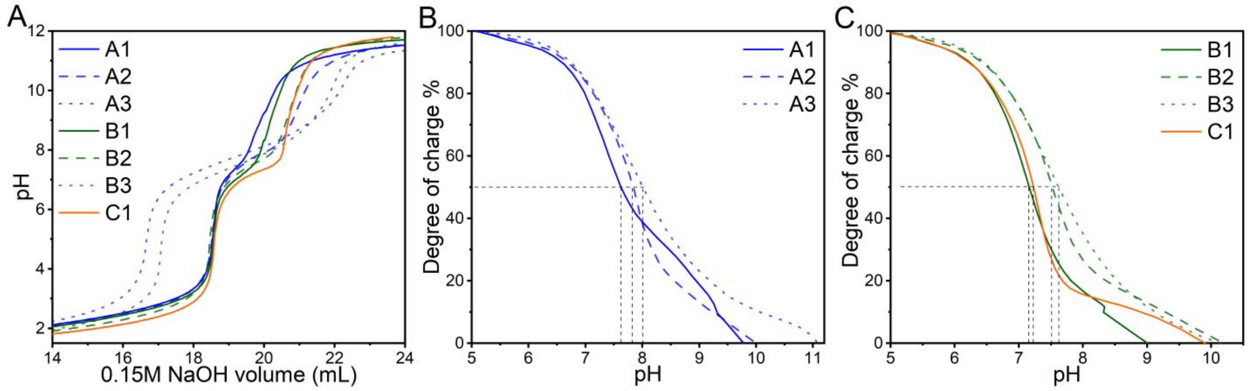

Figure S5. The titration curves of all polymers (A) for the polymer library. DoC of DMAEAm or DMAEA in the copolymers, determined using plots B and C.

Degradation of DMAEA

First, a 200 mM NaOH was first prepared by dissolving 40 mg NaOH in 5 mL D_2O. Generally, each polymer was dissolved in D_2O and a 200 mM NaOH was added to yield a final volume to 550 µL. The amounts are shown in the table below. ^{1}H NMR (300 MHz) of the solutions was measured at 64 scans and afterwards the samples were placed in an incubator at 37 ^oC for 24 h and measured again under the same conditions.

Table S2. Amounts used for preparation of ^{1}H NMR samples to analyze the self-hydrolysis of DMAEA.

Polymer code	Amount added (mg)	D_20 added (µL)	200 mM NaOH in D_2O added (µL)
**B2**	30.0	404	146
**B3**	30.0	220	330
**$PDMAEA_{125}$**	26.3	147	403

The ^{1}H NMR results are shown in Figure S6. The degradation was calculated using previously reported formula as shown in Equation S3.[5] A modified version (Equation S4) was used for the copolymers due to the overlapping of peak.

$$\% \text{ Degradation} = \frac{int\ a2}{int\ a1 + int\ a2} \cdot 100 \qquad \text{(Equation S3)}$$

$$\% \text{ Degradation} = \frac{int\ a_2}{\left(\frac{DP_{DMAEA}}{DP_{total}}\right) * int\ a_1 + int\ a_2} \cdot 100 \qquad \text{(Equation S4)}$$

Where int a_x is the integral peak of a_x as shown in Figure S6, DP_{DMAEA} and DP_{total} is the DP of DMAEA and total DP, respectively in polymer of interest.

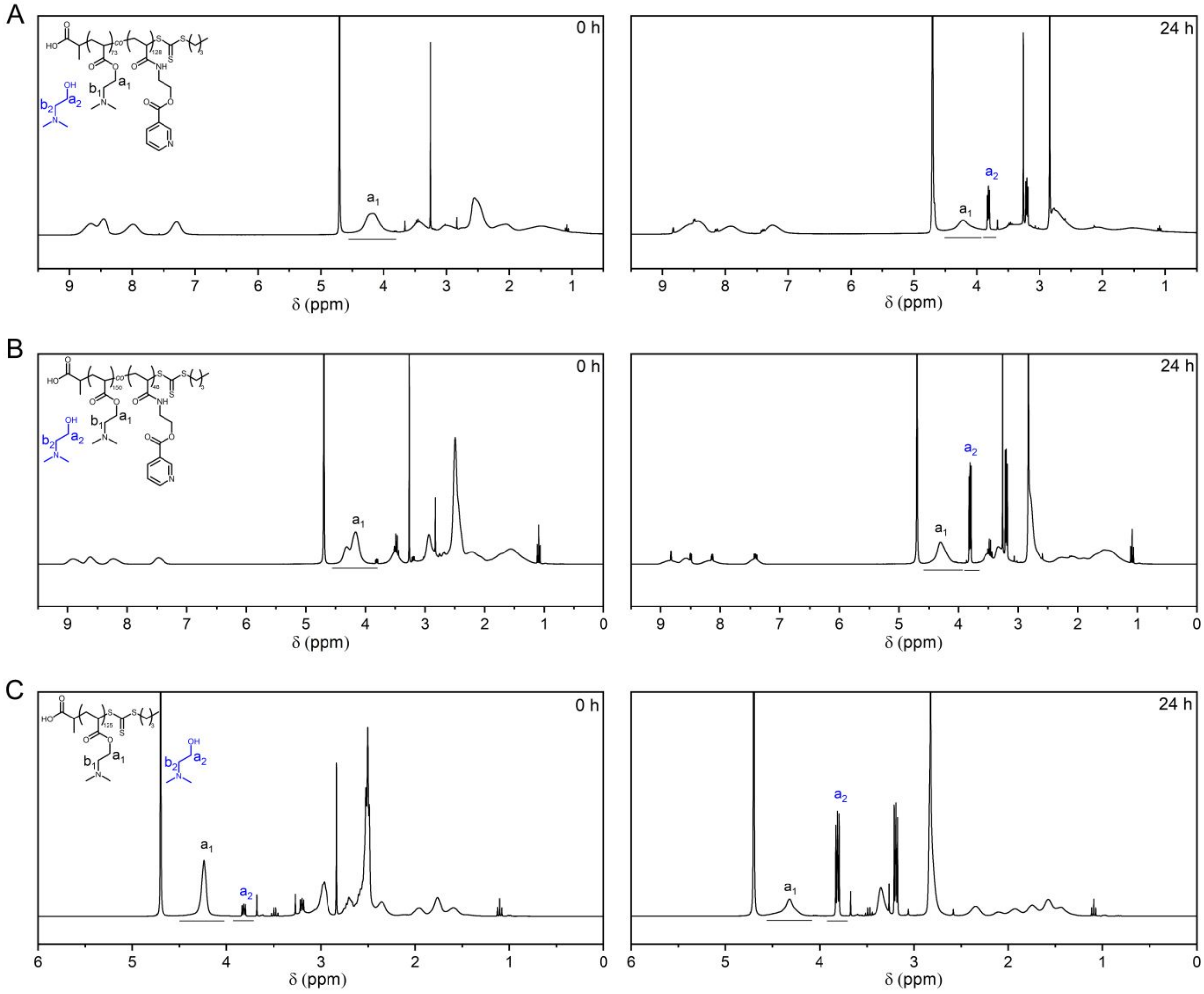

Figure S6. Degradation studies of DMAEA containing polymers, performed by ^{1}H NMR (300 MHz). Polymers B2 (A), B3 (B) and $PDMAEA_{125}$ were investigated at approx. pH 7.5, at the beginning (0 h) and after incubation at 37 ^{o}C for 24 h. B2, B3 and $PDMAEA_{125}$ had a degradation of 52%, 43% and 43% respectively. Minimal degradation at the beginning of the measurement was considered for the calculations.

N*/P ratio calculations

The N/P ratio was calculated according to a previously published protocol.[6] The N*/P ratio was defined as the ratio of the total amount of protonatable amines in polymer solution in relation to the total amount of phosphates in the pDNA solution. The volume of polymer needed to prepare polyplexes with 30 µg mL^{-1} pDNA at different N*/P ratios was calculated as described by the following equations:

$$V_{total} \cdot P = V_{poly} \cdot N_{poly}$$

$$V_{poly} = \frac{V_{total} \cdot P}{N_{poly}}$$

$$V_{poly} = V_{total} \cdot \frac{n_{pDNA} \cdot P}{n_{poly} \cdot N}$$

$$V_{poly} = V_{total} \cdot \frac{m_{pDNA} \cdot P \cdot M_{poly}}{m_{poly} \cdot N \cdot M_{pDNA}} \qquad \text{(Equation S5)}$$

Where V_{total}, P, V_{poly} and N_{poly} are the total required volume, the total number of phosphates of the pDNA, the required volume of polymer and the total number of active amines of the polymer, respectively.

Ethidium bromide quenching assay (EBA) and heparin dissociation assay (HRA)

Table S3. Kinetic cycle protocol for automated heparin addition by the microplate reader.

Kinetic cycle	Repetitions	Addition of heparin		Orbital shake	Incubation	Measurement
		V [µL]	Stock Solution / [U mL^{-1}]			
1	2	5	100	10 s	10 min, 37 °C	λEx = 525 nm / λEm = 605 nm
2	1	15	100	10 s	10 min, 37 °C	λEx = 525 nm / λEm = 605 nm
3	3	5	500	10 s	10 min, 37 °C	λEx = 525 nm / λEm = 605 nm
4	1	10	500	10 s	10 min, 37 °C	λEx = 525 nm / λEm = 605 nm

Further results

Heparin release assay (HRA)

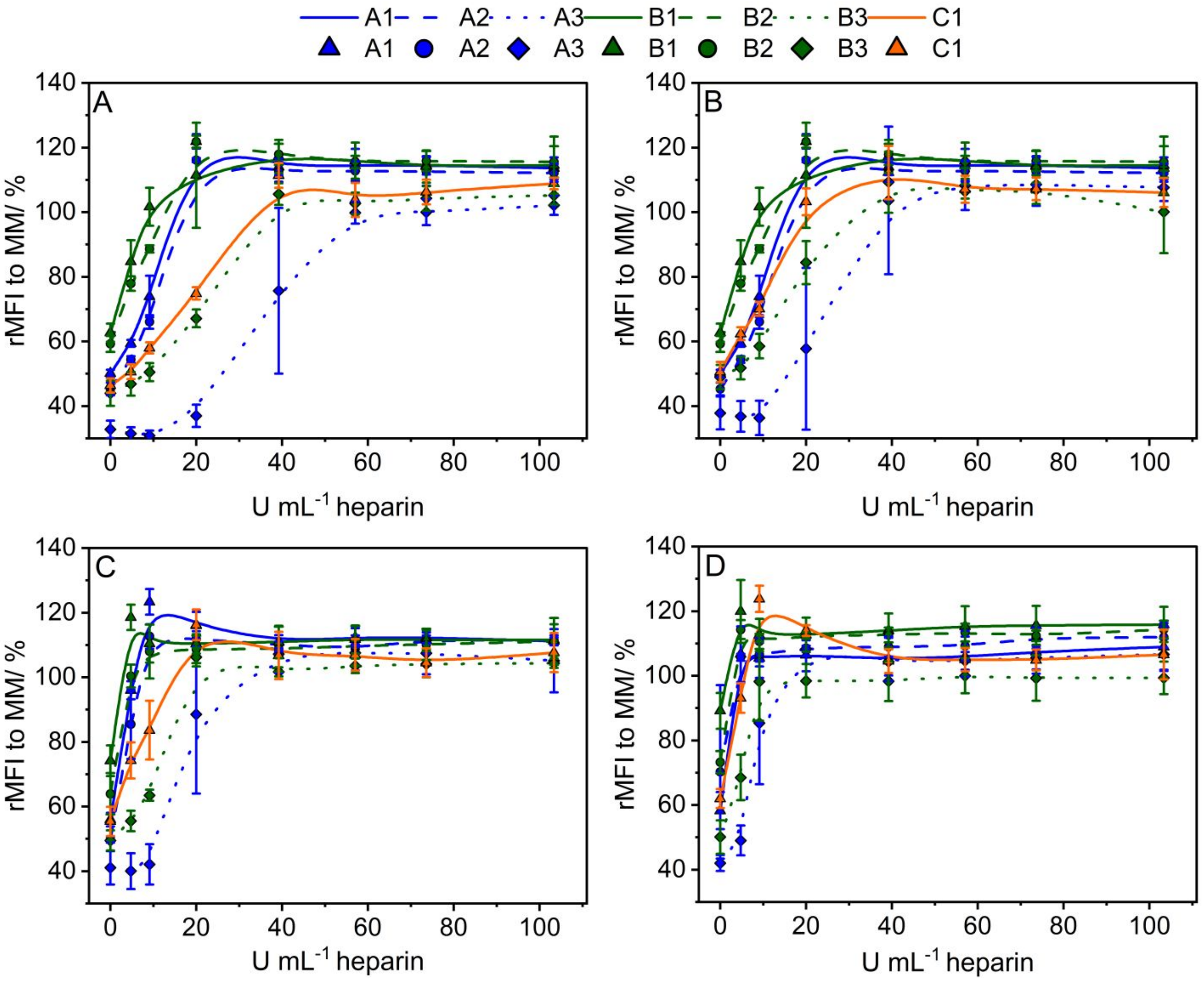

Figure S7. Heparin release assay. The increase of the fluorescence intensity correlates with the ability of the polymer to release the genetic material for **A**) N*/P 15, **B**) N*/P 10, **C**) N*/P 5, **D**) N*/P 3. Lines represent a fitted B-Spline function (n = 3).

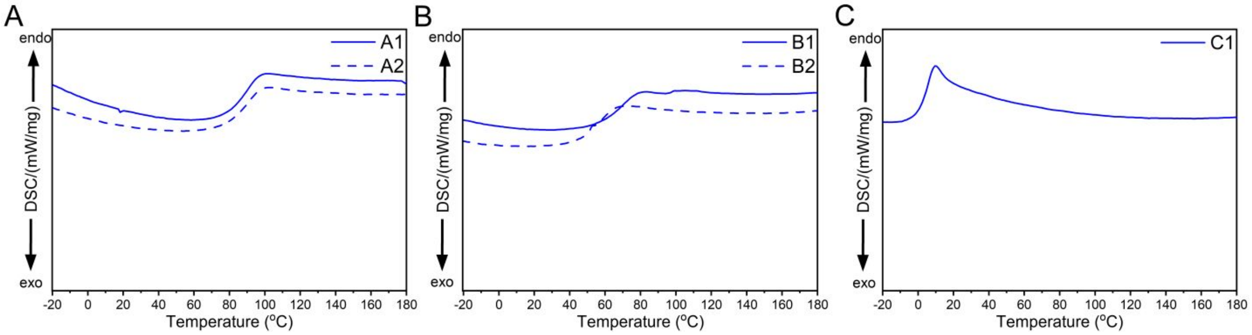

Figure S8. DSC plots display the glass transition temperature (T_g) for the polymers with the best transfection performance. The T_g was derived from the second heating cycle which was performed at 20 K min^{-1}. The software-calculated inflection points for each set ranged as follows: **A1**: 94 °C, **A2**: 96 °C, **B1**: 69 °C, **B2**: 52 °C and **C1**: 6 °C.

Cytocompatibility (PrestoBlue assay)

Table S4. Half maximal inhibitory concentrations IC_{50} determined via PrestoBlue assay in L929 cells over 24 h incubation. Polymer concentrations at each pDNA concentration are also provided.

Polymer	LPEI	A1	A2	A3	B1	B2	B3	C1
IC_{50}/ µg mL^{-1}	39	217	136	143	289	175	-	37
IC_{70}/ µg mL^{-1}	32	175	100	113	214	139	-	32
c (polymer) / µg mL^{-1} [c(pDNA) = 3 µg mL^{-1}]	7.7	153	99	47	150	94	46.2	106
c (polymer) / µg mL^{-1} [c(pDNA) = 2 µg mL^{-1}]	-	102	66	31	100	63	31	71
c (polymer) / µg mL^{-1} [c(pDNA) = 1 µg mL^{-1}]	-	51	33	16	50	31	15	35

Particle uptake study

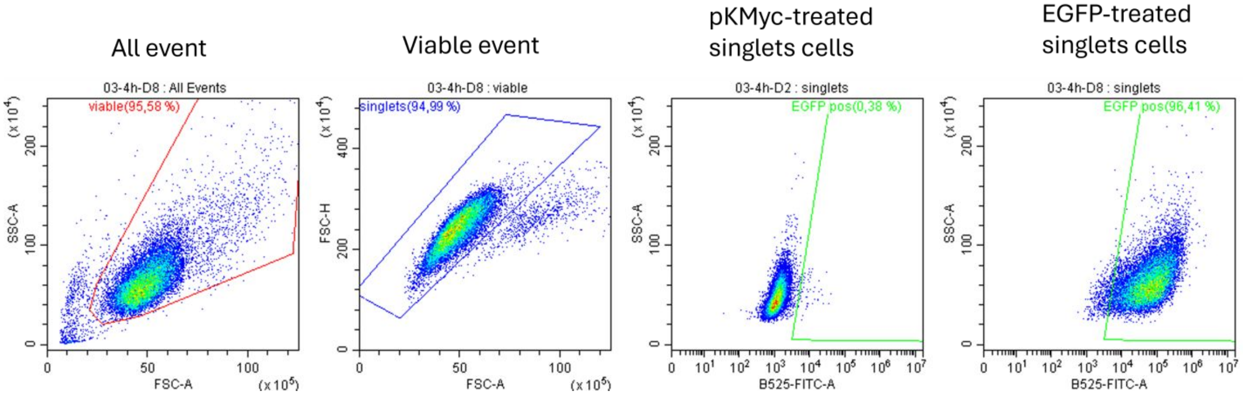

Figure S9. The gating strategy of the particle uptake study. For flow cytometry analysis 15 000 events were recorded in viable singlet gate (blue gate). The following strategy was used: Firstly, viable cells of all events were gated according to forward scatter/side scatter area pattern (FSC-A/SSC-A) (red gate). Viable cells were further gated in forward scatter high/forward scatter area pattern FCS-H/FSC-A (blue gate) to distinguish single cells to cell aggregation. Viable, single cells were gated to determine the YOYO-1 positive cells according to side scatter area/ bandpass detection filter 525 ± 40 nm (green gate). The YOYO-1 positive cells were gated using the same gate for the negative control (cells treated with pKMyc), where no polymer was used. Gating strategy is shown for B1 at N/P 20 and 3µg mL-1 pDNA after 4 h incubation.

Transfection efficiency

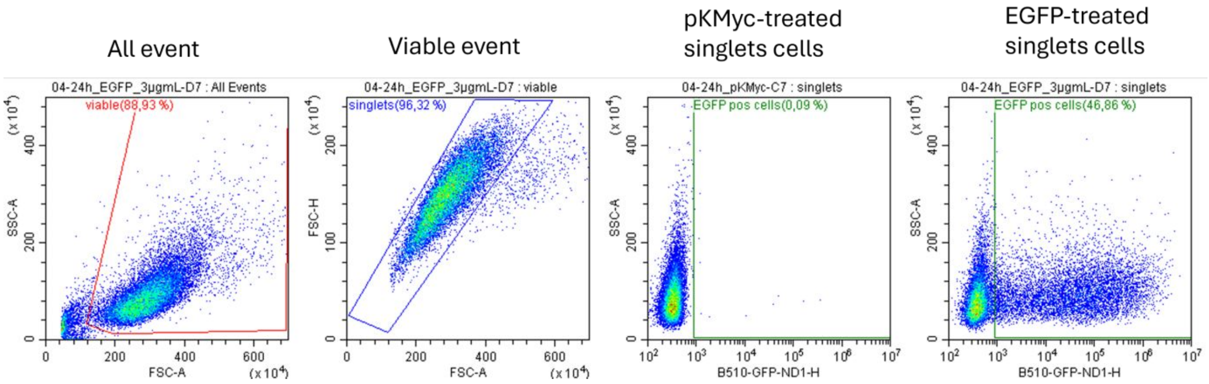

Figure S10. The gating strategy of the transfection efficiency. For flow cytometry analysis 15 000 events were recorded in viable singlet gate (blue gate). The following strategy was used: Firstly, viable cells of all events were gated according to forward scatter/side scatter area pattern (FSC-A/SSC-A) (red gate). Viable cells were further gated in forward scatter high/forward scatter area pattern FCS-H/FSC-A (blue gate) to distinguish single cells to cell aggregation. Viable, single cells were gated to determine the EGFP expression according to side scatter area/ bandpass detection filter 510 ± 10 nm with signal attenuation (OD1) high pattern (green gate) The EGFP expressing cells were gated using the same gate for the negative control (cells treated with pKMyc polyplexes), where no EGPF expression was possible. Gating strategy of pKMyc and pEGFP treated singlets cells were shown for B1 at N/P 20 and 3µg mL^{-1} pDNA.

Endosomal escape

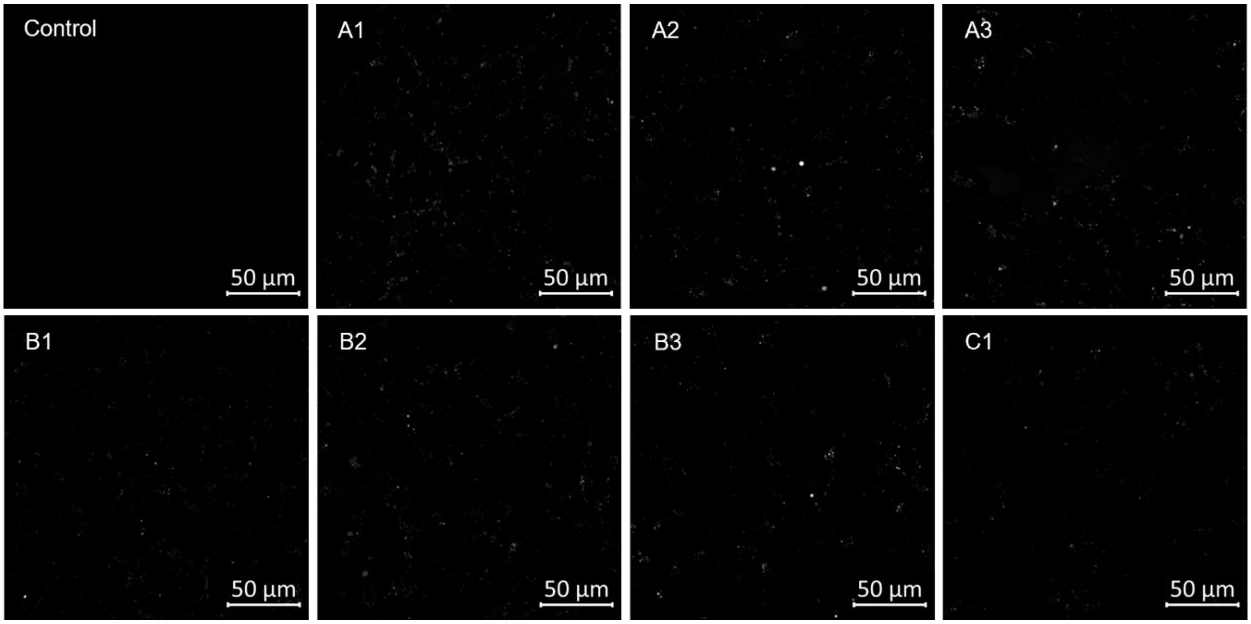

Figure S11. Endosomal release was analyzed via confocal laser scanning microscopy (CLSM) after 4 h. HEK293T cells were simultaneously incubated with non-permeable dye calcein (25 µg mL^{-1}) and polyplexes (N*/P 20, 3 µg mL^{-1} pDNA). Grey dots indicate endocytotic uptake of calcein within cellular compartments, and diffuse grey fluorescence pattern indicates endosomal calcein release.

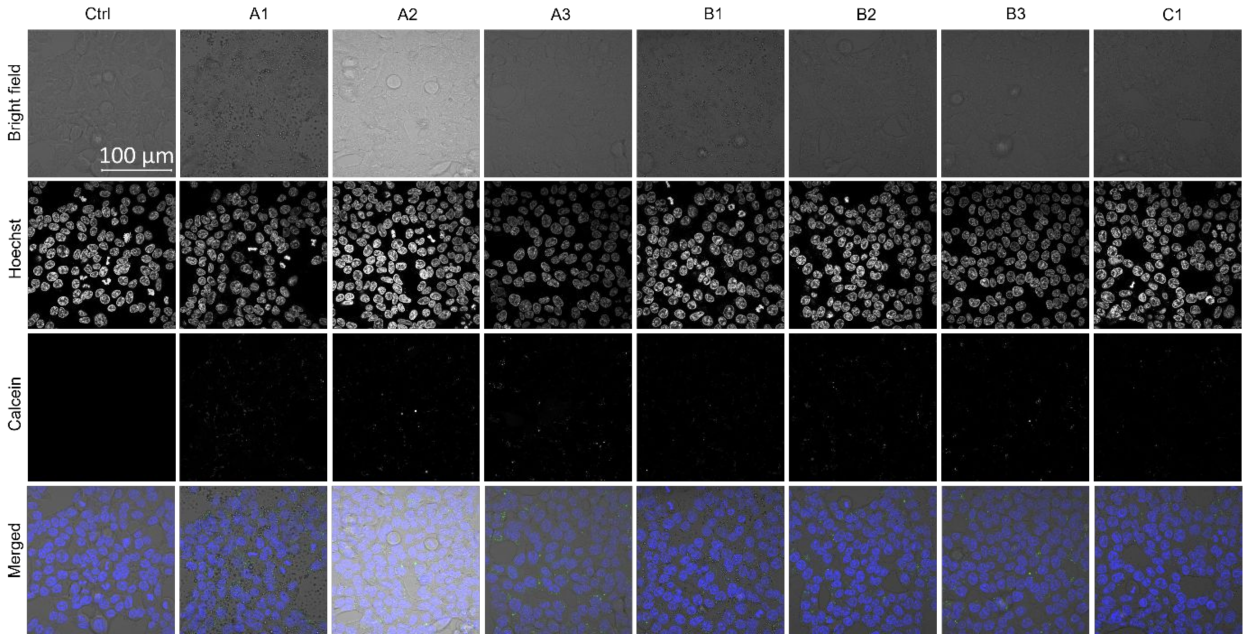

Figure S12. Endosomal release was analyzed via confocal laser scanning microscopy (CLSM) over 4 h in full growth medium with 10% serum. HEK293T cells were simultaneously incubated with non-permeable dye calcein (25 µg mL^{-1}) and polyplexes (N*/P 20, 3 µg mL^{-1} pDNA). In merged images, cell nuclei stained with Hoechst 33342 is blue and the calcein signal is green. The non-treated sample was used as the controls.

CytoTox-ONE assay at different pDNA concentrations

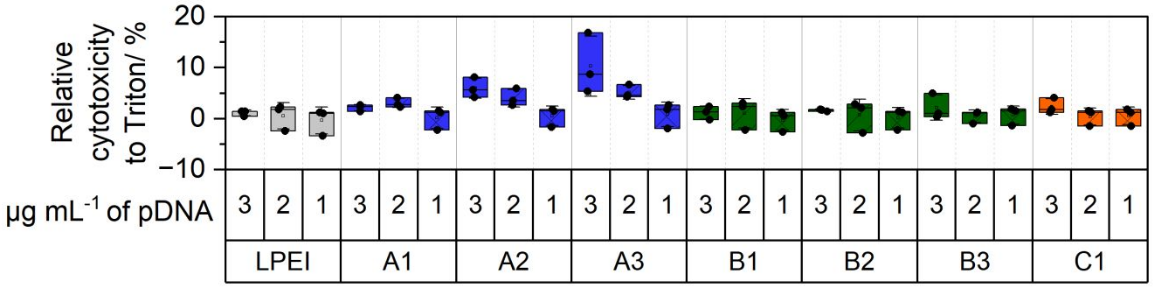

Figure S13. CytoTox-ONE assay of polyplexes (transfection efficiency assay) at different pDNA concentrations and N*/P 20 on HEK293T cells. Cells were incubated in full growth medium (D10H) over 24 h (n = 3).

Transfection efficiency

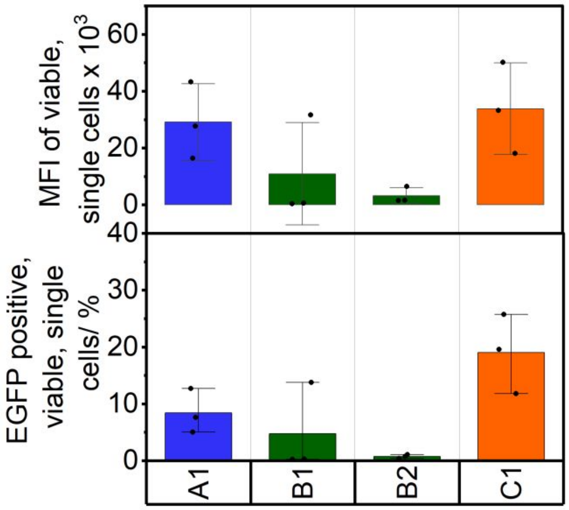

Figure S14. A closer look at the transfection efficiency of **A1**, **B1**, **B2** and **C1** polymers at 1 µg mL^{-1} pDNA.

CytoTox-ONE assay at different N*/P ratios

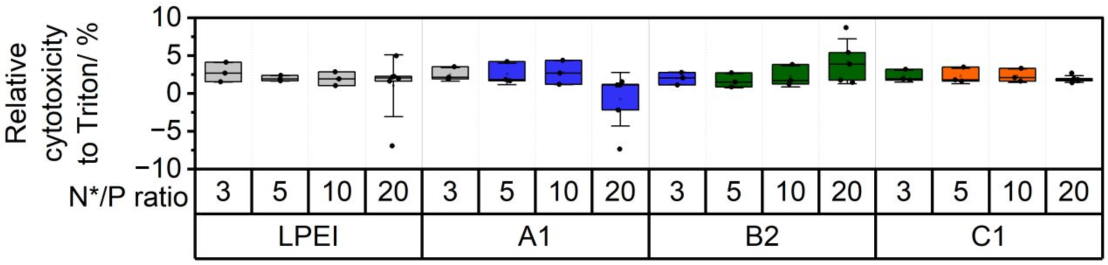

Figure S15. CytoTox-ONE assay of polyplexes (transfection efficiency assay) at different N*/P ratios and c(pDNA) = 3 µg mL^{-1} on HEK293T cells. Cells were incubated in full growth medium (D10H) over 24 h (n = 3).

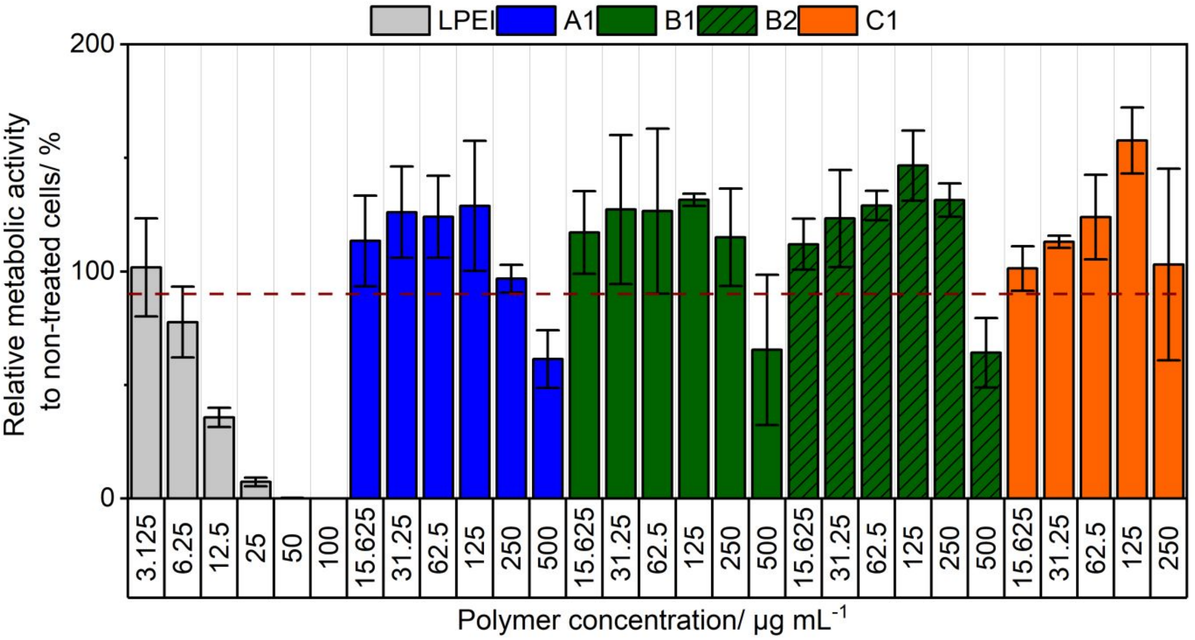

Figure S16. PrestoBlue assay of THP-1 cell lines over 24 h (n = 3).

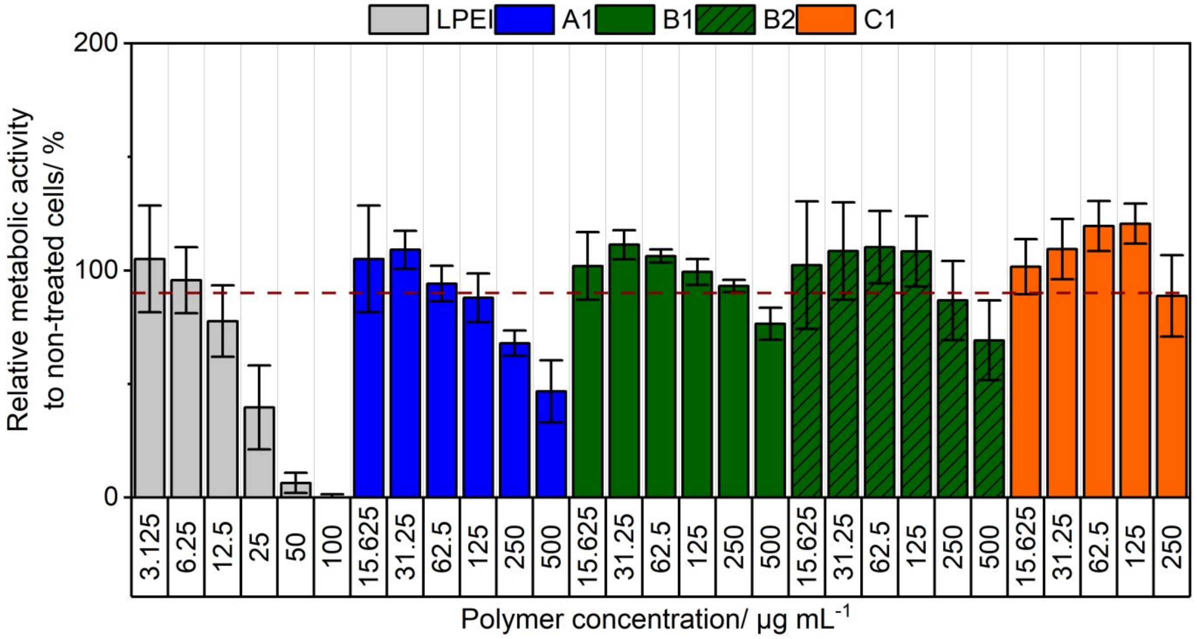

Figure S17. PrestoBlue assay of Jurkat cell lines over 24 h (n = 3).

Table S5: Antibody mix for murine monocyte staining.

Reagent	Volume per 1x
2% FBS	50ul
APC-Ly-6C antibody	1ul
PE-CD115 antibody	1.5ul
APC/Cyanine7-CD11b antibody	1.5ul

Table S6: Descriptive Statistics of EGFP positive cells in Figure 5B.

Polymer	Number of values	Mean	Std. Deviation	Std. Error of Mean
LPEI	3	0.09009	0.00847	0.00489
A1	3	0.652	0.09753	0.05631
A2	3	0.16569	0.02643	0.01526
A3	3	0.00782	0.00365	0.00211
B1	3	0.4536	0.18533	0.107
B2	3	0.44982	0.11596	0.06695
B3	3	0.00633	0.00295	0.0017
C1	3	0.55504	0.01838	0.01061

Table S7: One-Way ANOVA of EGFP positive cells with Turkey test was used for the means comparison of LPEI to all other used polymers in Figure 5B. Significance = 1 indicates that the difference in means comparison is significant at the respective alpha value.

Polymer	MeanDiff	Std. Error of Mean	q-value	Probability	Alpha	Significance
A1 LPEI	0.56191	0.06978	11.38731	1.12938E-5	0.001	1
A2 LPEI	0.0756	0.06978	1.53206	0.95148	0.001	0
A3 LPEI	-0.08227	0.06978	1.66716	0.92687	0.001	0
B1 LPEI	0.36351	0.06978	7.36667	0.00171	0.01	1
B2 LPEI	0.35973	0.06978	729011	0.0019	0.01	1
B3 LPEI	-0.08376	0.06978	169733	0.92047	0.001	0
C1 LPEI	0.46496	0.06978	9.42247	1.17093E-4	0.001	1

Table S8: Descriptive Statistics of EGFP positive cells in Figure 5C.

Polymer	c(pDNA) on cells/ µgmL-1	Number of values	Mean	Std. Deviation	Std. Error of Mean
LPEI	1	3	0.00902	0.00458	0.00264
A1	1	3	0.08487	0.03924	0.02265
A2	1	3	0.00396	0.00195	0.00113
A3	1	3	0.00422	0.00157	9,05E+01
B1	1	3	0.0476	0.07823	0.04517
B2	1	3	0.0074	0.00327	0.00189
B3	1	3	9.55556E-4	1.67774E-4	9,69E+00
C1	1	3	0.19162	0.07043	0.04066
LPEI	2	3	0.11931	0.02758	0.01592
A1	2	3	0.63798	0.05169	0.02984
A2	2	3	0.03027	0.00697	0.00402
A3	2	3	0.01876	0.00807	0.00466
B1	2	3	0.47087	0.17033	0.09834
B2	2	3	0.41391	0.24589	0.14196
B3	2	3	0.01058	4.2339E-4	2,44E+01
C1	2	3	0.62527	0.09524	0.05499
LPEI	3	3	0.35196	0.07737	0.04467
A1	3	3	0.67929	0.08268	0.04773
A2	3	3	0.20631	0.0426	0.02459
A3	3	3	0.03653	0.01704	0.00984
B1	3	3	0.55036	0.07664	0.04425
B2	3	3	0.63409	0.09518	0.05495
B3	3	3	0.01013	0.01009	0.00582
C1	3	3	0.60902	0.08078	0.04664

Table S9: One-Way ANOVA of EGFP positive cells with Turkey test was used for the means comparison of LPEI to all other used polymers in Figure 5C. Significance = 1 indicates that the difference in means comparison is significant at the respective alpha value.

Polymer	c(pDNA) on cells/ µgmL-1	MeanDiff	Std. Error of Mean	q-value	Probability	Alpha	Significance
A1 LPEI	1	0.07584	0.03248	3.30254	0.33342	0.05	0
A2 LPEI	1	-0.00507	0.03248	0.22062	1	0.05	0
A3 LPEI	1	-0.0048	0.03248	0.20901	1	0.05	0
B1 LPEI	1	0.03858	0.03248	1.67982	0.92422	0.05	0
B2 LPEI	1	-0.00162	0.03248	0.07064	1	0.05	0
B3 LPEI	1	-0.00807	0.03248	0.35125	0.99999	0.05	0
C1 LPEI	1	0.1826	0.03248	7.95107	7.80493E-4	0.001	1
A1 LPEI	2	0.51867	0.09224	7.95251	7.78993E-4	0.001	1
A2 LPEI	2	-0.08904	0.09224	1.36528	0.97328	0.05	0
A3 LPEI	2	-0.10056	0.09224	154178	0.94993	0.05	0
B1 LPEI	2	0.35156	0.09224	539026	0.02574	0.05	1
B2 LPEI	2	0.2946	0.09224	451699	0.08194	0.05	0
B3 LPEI	2	-0.10873	0.09224	166717	0.92687	0.05	0
C1 LPEI	2	0.50596	0.09224	775762	0.00101	0.01	1
A1 LPEI	3	0.32733	0.05514	839526	43,4666	0.001	1
A2 LPEI	3	-0.14564	0.05514	373541	0.21077	0.05	0
A3 LPEI	3	-0.31542	0.05514	808977	64,9408	0.01	1
B1 LPEI	3	0.1984	0.05514	508845	0.0387	0.05	1
B2 LPEI	3	0.28213	0.05514	7236	0.00204	0.01	1
B3 LPEI	3	-0.34182	0.05514	876686	26,8607	0.001	1
C1 LPEI	3	0.25707	0.05514	65931	0.00492	0.01	1

Table S10: Descriptive Statistics of MFI in Figure 5C.

Polymer	c(pDNA) on cells/ µgmL-1	Number of values	Mean	Std. Deviation	Std. Error of Mean
LPEI	3	3	135169.6	32250.54703	18619.86201
A1	3	3	381333.06667	84235.73453	48633.52401
A2	3	3	141508.36667	47728.13739	27555.85297
A3	3	3	7079.33333	1944.15108	1122.45615
B1	3	3	171579.73333	110338.0951	63703.72891
B2	3	3	424398.3	88017.94895	50817.18652
B3	3	3	6339.16667	7301.35024	4215.43653
C1	3	3	282890.63333	34726.60039	20049.41209

Table S11: One-Way ANOVA of MFI with Turkey test was used for the means comparison of LPEI to all other used polymers in Figure 5C. Significance = 1 indicates that the difference in means comparison is significant at the respective alpha value.

Polymer	c(pDNA) on cells/ µgmL-1	MeanDiff	Std. Error of Mean	q-value	Probability	Alpha	Significance
A1 LPEI	3	246163.46667	51314.7255	6.78417	0.00379	0.01	1
A2 LPEI	3	6338.76667	51314.7255	0.17469	1	0.05	0
A3 LPEI	3	-128090.26667	51314.7255	3.53012	0.26386	0.05	0
B1 LPEI	3	36410.13333	51314.7255	1.00345	0.99541	0.05	0
B2 LPEI	3	289228.7	51314.7255	7.97103	7.60065E-4	0.001	1
B3 LPEI	3	-128830.43333	51314.7255	3.55052	0.25818	0.05	0
C1 LPEI	3	147721.03333	51314.7255	4.07113	0.1426	0.05	0

Table S12: Descriptive Statistics of EGFP positive cells in Figure 6.

Polymer	N*/P ratio	Number of values	Mean	Std. Deviation	Std. Error of Mean
LPEI	3	3	6.88889E-4	1.38778E-4	8.01234E-5
A1	3	3	0.00253	8.35331E-4	4.82279E-4
B2	3	3	0.00458	0.00505	0.00292
C1	3	3	0.01071	0.00829	0.00478
LPEI	5	3	0.00178	2.34126E-4	14
A1	5	3	0.07656	0.03125	0.01804
B2	5	3	0.00124	0.00117	68
C1	5	3	0.05731	0.02443	0.0141
LPEI	10	3	0.04482	0.01077	0.00622
A1	10	3	0.55862	0.13183	0.07611
B2	10	3	0.16551	0.16895	0.09754
C1	10	3	0.56764	0.20969	0.12107

Table S13: One-Way ANOVA of EGFP positive cells with Turkey test was used for the means comparison of LPEI to all other used polymers in Figure 6. Significance = 1 indicates that the difference in means comparison is significant at the respective alpha value.

Polymer	N*/P ratio	MeanDiff	Std. Error of Mean	q-value	Probability	Alpha	Significance
A1 LPEI	3	0.00184	0.00398	0.6559	0.96494	0.05	0
B2 LPEI	3	0.00389	0.00398	138292	0.76554	0.05	0
C1 LPEI	3	0.01002	0.00398	356399	0.1307	0.05	0
A1 LPEI	5	0.07478	0.0162	65278	0.00746	0.01	1
B2 LPEI	5	-53,3333	0.0162	0.04656	0.99999	0.05	0
C1 LPEI	5	0.05553	0.0162	484784	0.03645	0.05	1
A1 LPEI	10	0.5138	0.12248	593247	0.01285	0.05	1
B2 LPEI	10	0.12069	0.12248	139351	0.7616	0.05	0
C1 LPEI	10	0.52282	0.12248	603664	0.01167	0.05	1

Table S14: Descriptive Statistics of MFI in Figure 6.

Polymer	N*/P ratio	Number of values	Mean	Std. Deviation	Std. Error of Mean
LPEI	3	3	342.96667	26.16951	15.10897
A1	3	3	1314.03333	334.00673	192.83887
B2	3	3	3813.1	3289.14476	1898.98861
C1	3	3	7433.7	5544.64573	3201.20271
LPEI	5	3	508.03333	101.19893	58.42723
A1	5	3	63339.13333	25202.74305	14550.81048
B2	5	3	970.13333	885.42931	511.20285
C1	5	3	10840.66667	3461.07785	1998.25423
LPEI	10	3	24034.96667	8157.1005	4709.50417
A1	10	3	408390.33333	87731.70452	50651.92322
B2	10	3	144056.1	149864.91951	86524.55162
C1	10	3	321419.53333	152912.0595	88283.81871

Table S15: One-Way ANOVA of MFI with Turkey test was used for the means comparison of LPEI to all other used polymers in Figure 6. Significance = 1 indicates that the difference in means comparison is significant at the respective alpha value.

Polymer	N*/P ratio	MeanDiff	Std. Error of Mean	q-value	Probability	Alpha	Significance
A1 LPEI	3	971.06667	2635.45742	0.52108	0.98171	0.05	0
B2 LPEI	3	3470.13333	2635.45742	1.86211	0.57847	0.05	0
C1 LPEI	3	7090.73333	2635.45742	3.80496	0.10295	0.05	0
A1 LPEI	5	62831.1	10391.91627	8.55055	0.00138	0.01	1
B2 LPEI	5	462.1	10391.91627	0.06289	0.99997	0.05	0
C1 LPEI	5	10332.63333	10391.91627	1.40615	0.75687	0.05	0
A1 LPEI	10	384355.36667	94520.70523	5.7507	0.01524	0.05	1
B2 LPEI	10	120021.13333	94520.70523	1.79575	0.60456	0.05	0
C1 LPEI	10	297384.56667	94520.70523	4.44945	0.05411	0.05	0

Table S16: Descriptive Statistics of Data in Figure 7A regarding EGFP pos cells.

Polymer	Number of values	Mean	Std. Deviation	Std. Error of Mean
LPEI	3	0.03179	0.01021	0.0059
A1	3	0.04423	0.04459	0.02574
B2	3	0.01572	0.00876	0.00506
C1	3	0.05564	0.04195	0.02422

Table S17: Descriptive statistics of data in Figure 7B.

Treatment	Non-treated	Buffer	Niacin	Polymer C1	pDNA	Polyplex
Number of values	11	12	12	11	12	11
Minimum	0.8400	0.8000	0.5200	0.2800	0.5700	0.3800
Maximum	1.310	1.550	1.740	1.250	1.890	1.807
Range	0.4700	0.7500	1.220	0.9700	1.320	1.427
Mean	1.009	1.119	1.032	0.8010	1.037	1.127
Std. Deviation	0.1389	0.2600	0.3339	0.2852	0.3559	0.4441
Std. Error of Mean	0.04188	0.07505	0.09639	0.08600	0.1027	0.1339

REFERENCES

(1) Larnaudie, S. Supramolecular cyclic peptide-polymer nanotubes as drug delivery vectors. University of Warwick, 2017.

(2) Haag, S. M.; Murthy, A. Murine monocyte and macrophage culture. *Bio-protocol* **2021**, *11* (6), e3928-e3928.

(3) Mapfumo, P. P.; Solomun, J. I.; Becker, F.; Moek, E.; Leiske, M. N.; Rudolph, L. K.; Brendel, J. C.; Traeger, A. Vitamin B3 Containing Polymers for Nanodelivery. *Macromol. Biosci.* **2024**, 2400002. DOI: 10.1002/mabi.202400002.

(4) Catrouillet, S.; Brendel, J. C.; Larnaudie, S.; Barlow, T.; Jolliffe, K. A.; Perrier, S. Tunable length of cyclic peptide–polymer conjugate self-assemblies in water. *ACS Macro Lett.* **2016**, *5* (10), 1119-1123. DOI: 10.1021/acsmacrolett.6b00586.

(5) Truong, N. P.; Jia, Z.; Burges, M.; McMillan, N. A.; Monteiro, M. J. Self-catalyzed degradation of linear cationic poly (2-dimethylaminoethyl acrylate) in water. *Biomacromolecules* **2011**, *12* (5), 1876-1882. DOI: 10.1021/bm200219e.

(6) Richter, F.; Martin, L.; Leer, K.; Moek, E.; Hausig, F.; Brendel, J. C.; Traeger, A. Tuning of Endosomal Escape and Gene Expression by Functional Groups, Molecular Weight and Transfection Medium: A Structure-Activity Relationship Study. *J. Mater. Chem B.* **2020**. DOI: 10.1039/D0TB00340A.
